# Liposomal and Micellar-Based Nanoformulations of Fluorinated Curcumin Derivative and Naringenin—Comparative Studies

**DOI:** 10.3390/nano16150920

**Published:** 2026-07-27

**Authors:** Joanna Kuzminska, Agnieszka Sobczak, Ludwika Piwowarczyk, Violetta Krajka-Kuźniak, Rafał Pietrzyk, Mikołaj Baranowski, Paweł Bilski, Aneta Woźniak-Braszak, Tomasz Goslinski, Anna Jelińska

**Affiliations:** 1 Poznan University of Medical Sciences, Chair and Department of Pharmaceutical Chemistry, Rokietnicka 3, 60-806 Poznan, Poland; lpiwowarczyk@ump.edu.pl (L.P.); ajelinska@ump.edu.pl (A.J.); 2 Doctoral School, Poznan University of Medical Sciences, Bukowska 70, 60-812 Poznan, Poland; 3 Poznan University of Medical Sciences, Chair and Department of Pharmaceutical Biochemistry, Rokietnicka 3, 60-806 Poznan, Poland; vkrajka@ump.edu.pl; 4 Functional Materials Physics Division, Faculty of Physics and Astronomy, Adam Mickiewicz University, Uniwersytetu Poznanskiego 2, 61-614 Poznan, Poland; rafal.pietrzyk@amu.edu.pl (R.P.); mikbar@amu.edu.pl (M.B.); aneta.wozniak-braszak@amu.edu.pl (A.W.-B.); 5 Department of Experimental Physics of Condensed Phase, Faculty of Physics and Astronomy, Adam Mickiewicz University, Uniwersytetu Poznanskiego 2, 61-614 Poznan, Poland; bilski@amu.edu.pl; 6 Poznan University of Medical Sciences, Chair and Department of Chemical Technology of Drugs, Rokietnicka 3, 60-806 Poznan, Poland; tomasz.goslinski@ump.edu.pl

**Keywords:** fluorinated curcumin derivative, naringenin, prostate cancer, bladder cancer, polymeric micelles, liposomes, drug nanocarrier

## Abstract

**Background/Objectives**: Poor aqueous solubility and low bioavailability limit the therapeutic use of many hydrophobic anticancer agents. This study developed liposomal and polymeric micellar formulations of a fluorinated curcumin derivative (FCur) and naringenin (NG), prepared as single-compound and mixed systems, and compared their physicochemical and biological properties. **Methods**: Soluplus^®^-based polymeric micelles and POPC:DOTAP liposomes were prepared by the thin-film hydration method and characterised using dynamic light scattering (DLS), zeta potential measurements, HPLC, NMR relaxation studies, and in vitro cytotoxicity assays. **Results**: Polymeric micelles formed homogeneous dispersions with particle sizes below 95 nm and a slightly negative zeta potential (~−3 mV), whereas liposomes were larger (>130 nm) and strongly positively charged (>+40 mV). Both systems achieved high encapsulation efficiencies (>72% for FCur and >95% for NG). NMR relaxation studies revealed more restricted molecular dynamics within liposomal bilayers and greater motional freedom in micelles. In biological studies, FCur and mixed liposomes exhibited the highest overall cytotoxicity against bladder (5637), prostate (LNCaP) cancer as well as normal fibroblast (MRC-5) cell lines, compared with the free compounds and polymeric micelles. Notably, polymeric micelles with FCur (single and mixed) exhibited a more favourable differential cytotoxicity response between bladder cancer and normal cells. **Conclusions**: These findings demonstrate that the nanocarrier system plays a critical role in determining both molecular dynamics and biological performance of encapsulated agents.

## 1. Introduction

Bladder and prostate cancers remain among the most frequently diagnosed malignancies and leading causes of cancer-related mortality in men worldwide. According to recent global cancer statistics, prostate cancer was the second most commonly diagnosed cancer in men in 2024, accounting for approximately 1.5 million new cases and nearly 400,000 deaths worldwide. In the same year, bladder cancer accounted for more than 610,000 newly diagnosed cases and approximately 220,000 deaths globally, remaining one of the most common urological malignancies associated with high recurrence rates and substantial clinical burden [1,2,3]. Despite significant progress in cancer diagnostics and therapy, there remains a pressing need for novel anticancer agents and therapeutic strategies that offer improved efficacy, enhanced pharmacokinetic properties (including increased bioavailability and more efficient tumor delivery), a more favourable safety profile, greater selectivity toward cancer cells, and the ability to overcome multidrug resistance [4].

**Figure 1:**
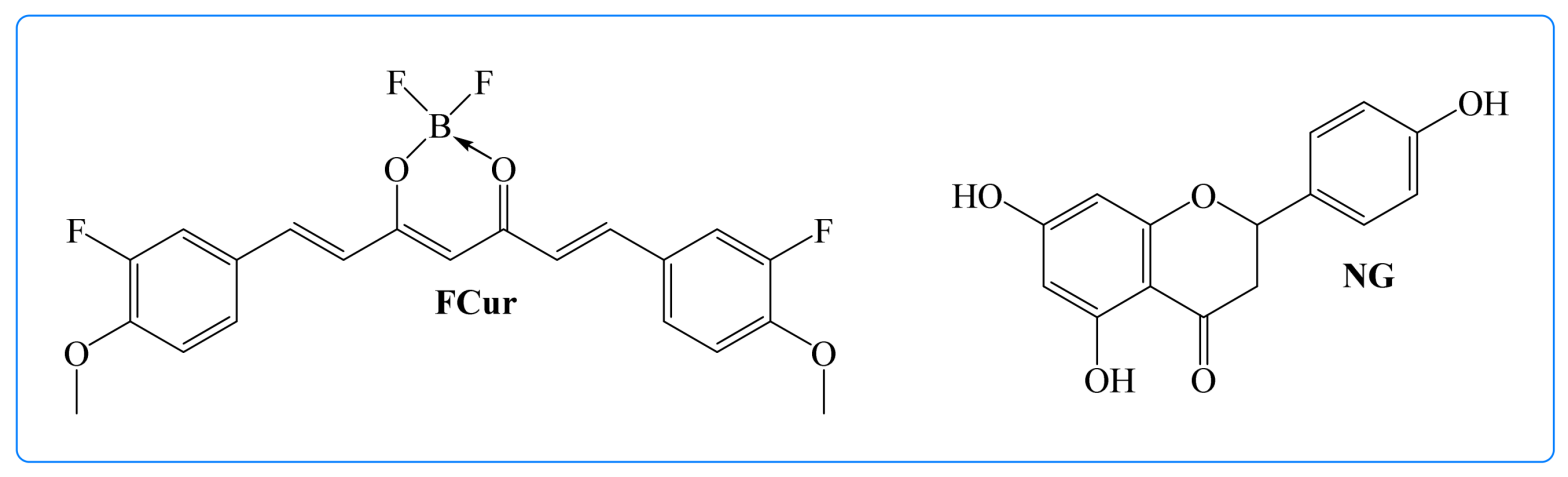
Chemical structures of fluorocurcumin derivative (FCur) and naringenin (NG).

In this context, accumulating evidence suggests that in prostate and bladder cancer models, various natural compounds, including polyphenols such as quercetin, resveratrol, curcumin, and naringenin (NG), show promising anticancer activity (Figure 1) [5,6,7,8,9,10,11]. Naringenin, and in particular curcumin and its synthetic analogues, have been extensively investigated due to their ability to modulate multiple signaling pathways associated with cancer progression and apoptosis, and to inhibit cancer cell proliferation, cell migration, and multidrug resistance [12,13,14,15,16,17]. In both prostate and bladder cancer models, curcumin has been reported to exert antiproliferative and proapoptotic effects, and to inhibit cancer cell migration and invasion through the regulation of multiple oncogenic signaling pathways, such as PI3K/Akt/mTOR, NF-κB, and STAT3 [10,18]. In turn, NG has previously been shown to reduce the viability and proliferation of bladder cancer cells (e.g., TSGH-8301) [13], and prostate cancer cells. In the latter case, the effect of NG on inhibiting proliferation and inducing apoptosis in PC3 and LNCaP prostate cancer cells was studied at concentrations ranging from 25 to 300 μM, demonstrating a cytotoxic effect at higher concentrations (>100 μM) [19].

Growing evidence indicates that combining natural polyphenols or co-administering them with conventional chemotherapeutic agents can enhance anticancer efficacy beyond the effects of single-agent treatments. For instance, curcumin (CUR) combined with quercetin has demonstrated synergistic antiproliferative and pro-apoptotic effects in prostate cancer models (PC-3 cells), resulting in stronger inhibition of cell growth than either compound alone [20]. Likewise, mixtures of curcuminoids, including curcumin, demethoxycurcumin, and bisdemethoxycurcumin, have shown synergistic anticancer activity in prostate cancer cells [21]. In bladder cancer models, curcumin co-treatment with cisplatin significantly increased apoptosis and growth inhibition, while its combination with chemotherapeutics such as doxorubicin, paclitaxel, or docetaxel has been reported across prostate and bladder cancer models, often associated with reduced drug resistance and improved therapeutic outcomes [22,23].

Similar observations have been reported for naringenin-based combination strategies, suggesting that polyphenol-based co-treatment approaches may represent a promising area for improving anticancer efficacy. NG has been shown to enhance the antitumor activity of doxorubicin in cancer models, including breast cancer and leukemia, by promoting apoptosis and partially overcoming multidrug resistance through modulation of MRP transporters [24]. In addition, NG combined with quercetin has demonstrated synergistic antiproliferative effects in non-prostate solid tumor models, in breast cancer cell lines [25]. Taken together, these findings suggest that NG and CUR are promising bioactive compounds; however, their therapeutic applicability remains limited by poor aqueous solubility, low bioavailability, rapid metabolism, and insufficient physicochemical stability under physiological conditions [26,27,28].

Accordingly, numerous formulation strategies have been developed to overcome the above limitations, and to improve the physicochemical and biological properties of naringenin, curcumin, and synthetic curcumin analogues. Previous studies have investigated various polymer- and liposome-based formulations for CUR delivery. PLGA nanospheres containing CUR prepared by a solvent evaporation method exhibited high encapsulation efficiency, sustained drug release and enhanced cellular uptake, resulting in improved antiproliferative activity compared with free compound in LNCaP, PC3 and DU145 prostate cancer cell lines. In parallel, Soluplus^®^-based polymeric micelles with CUR prepared by a solid-dispersion approach significantly improved the aqueous solubility and stability of curcumin while maintaining its anticancer activity in NCI-H520 lung squamous carcinoma cells. Liposomal formulations have also been extensively studied as CUR drug delivery systems. For example, phospholipid-based PEGylated liposomes, such as DSPE-PEG2000, have demonstrated high encapsulation efficiency, increased stability in plasma, and enhanced antitumor activity against pancreatic adenocarcinoma cell lines (AsPC-1, BxPC-3, and PANC-1) [29,30,31]. Soluplus^®^ has attracted increasing interest in the pharmaceutical industry due to its biocompatibility, high solubilization capacity, and ability to stabilise amorphous drug dispersions [32,33,34]. In contrast, DOTAP-containing liposomes facilitate intracellular delivery of encapsulated agents [35,36]. Both systems can enhance apparent solubility, protect labile compounds, and modulate drug-cell interactions. However, considerably less attention has been devoted to understanding how local molecular organisation and molecular dynamics within nanocarrier systems affect intermolecular interactions, drug mobility, and, ultimately, the biological activity of encapsulated compounds.

To overcome some of the well-known limitations of natural curcumin, including its limited stability and biological activity, our previous research has focused on developing fluorinated curcumin analogues with enhanced pharmacological potential. As part of this research, a series of fluorinated curcumin analogues was synthesized, and their biological activity was assessed. Among them, the compound (1*E*,4*Z*,6*E*)-5-((difluoroboryl)oxy)-1,7-bis(3-fluoro-4-methoxyphenyl)hepta-1,4,6-trien-3-one (FCur; Figure 1) exhibited the strongest cytotoxic activity after 24 h of incubation against the 5637 and SCaBER bladder cancer cell lines, with markedly lower IC_50_ values than native curcumin (FCur: 6.49 and 3.31 μM vs. CUR: 12.65 and 13.14 μM, respectively) [14,37]. Similarly, NG, as mentioned before, exhibits anticancer activity against bladder and prostate cancer cells.

Guided by the above considerations, FCur and NG were selected as promising candidates for further investigation. Instead of native curcumin, we employed our synthesized fluorinated curcumin derivative, which had previously demonstrated superior cytotoxicity against prostate and bladder cancer cells compared with the parent compound. Since previous studies have suggested that co-administration of polyphenols may provide additional therapeutic benefits, both compounds were evaluated individually and in combination. To improve their delivery, liposomal and polymeric nanoformulations were developed for FCur, NG, and their mixture. The parallel development and direct comparison of two nanocarrier platforms encapsulating the same individual compounds, as well as their combination, remain rarely explored. Therefore, the aim of this study was to develop and comprehensively characterize these nanoformulations and to investigate the relationships among the type of nanocarrier, physicochemical properties, cytotoxic activity against prostate and bladder cancer cells, as well asnormal fibroblast cells, and molecular dynamics as determined by NMR relaxation studies. Additionally, the micellar formulations were freeze-dried to assess the effect of lyophilization on their physicochemical properties and molecular behaviour.

## 2. Materials and Methods

### 2.1. Chemical Compounds and Reagents

Fluorocurcumin derivative (FCur) (1*E*,4*Z*,6*E*)-5-((difluoroboryl)oxy)-1,7-bis(3-fluoro-4-methoxyphenyl)hepta-1,4,6-trien-3-one (420.16 g/mol) was prepared according to a literature procedure [37], purity of 98.7% was determined by HPLC. Naringenin (NG), (±)-2,3-dihydro-5,7-dihydroxy-2-(4-hydroxyphenyl)-4H-1-benzopyran-4-one (272.26 g/mol), acetonitrile (ACN) for HPLC, and chloroform for the preparation of stock solutions, were obtained from Sigma-Aldrich (St. Louis, MO, USA). 1-Palmitoyl-2-oleoyl-glycero-3-phosphocholine (POPC) and 1,2-dioleoyl-3-trimethylammonium-propane (DOTAP) were obtained from Avanti Polar Lipids (Birmingham, AL, USA). Soluplus^®^, poly(N-vinylcaprolactam)-poly(vinyl acetate)-poly(ethylene glycol), was provided kindly by BASF of Ludwigshafen, Germany. Acetic acid and ethanol were obtained from Avantor Performance Materials (Gliwice, Poland). Sterile water for injection (Aqua ad injectabilia) was obtained from B. Braun, Melsungen, Germany. Reagents used for biological activity (MTT assay), such as RPMI-1640 medium, Dulbecco’s Modified Eagle’s Medium (DMEM), fetal bovine serum (FBS), phosphate-buffered saline (PBS), trypsin-EDTA, L-glutamine, dimethylsulfoxide (DMSO), 3-(4,5-dimethylthiazol-2-yl)-2,5-diphenyltetrazolium bromide (MTT), were obtained from Sigma Aldrich, St. Louis, MO, USA.

### 2.2. Liposome Preparation

The lipids used in the study were dissolved in chloroform, while the compounds (FCur and NG) were dissolved in acetonitrile. Liposomes were prepared from 1-palmitoyl-2-oleoyl-sn-glycero-3-phosphocholine (POPC) and 1,2-dioleoyl-3-trimethylammonium-propane (DOTAP) mixture (8:0.8) (LPs), and either FCur (FCur-LPs 8:0.8:0.1), NG (NG-LPs 8:0.8:0.1), or their combination (MIX-LPs) with the following molar ratio (MIX-LPs 8:0.8:0.05:0.05). The final concentrations were as follows: 12.16 mg/mL POPC, 55.88 µg/mL DOTAP, and 27.2 µg/mL (100 µM) NG in NG-LPs, or 42.0 µg/mL (100 µM) FCur in FCur-LPs, or 21.0 µg/mL (50 µM) of FCur and 13.6 µg/mL (50 µM) of NG in MIX-LPs, respectively.

The solvents from the samples were evaporated under reduced pressure using high-vacuum rotary evaporation for 20 min to form a dry lipid film, which was hydrated in water for injections, sonicated for 9 to 12 min to obtain liposomes.

### 2.3. Micelle Preparation

Soluplus^®^ stock solutions of appropriate concentrations were prepared in dry ethanol, while the investigated compounds were dissolved in ACN. Appropriate volumes of these stock solutions were then transferred into a round-bottom flask, mixed thoroughly, and the solvents were evaporated under reduced pressure using a rotary evaporator (100 rpm, 10 min, 25 °C) to obtain a thin film. The resulting thin film was subsequently hydrated with 5.0 mL of water for injection on a rotary evaporator at 100 rpm for 10 min, followed by an additional 30 min of shaking (300 rpm). The final micellar dispersions contained Soluplus^®^ at concentrations of 31.50 mg/mL and 27.2 µg/mL (100 µM) NG in NG-PMs, or 42.0 µg/mL (100 µM) FCur in FCur-PMs, or 21.0 µg/mL (50 µM) FCur with 13.6 µg/mL (50 µM) NG in MIX-PMs. In the next step, the formulation was filtered through a 0.22 µm PTFE syringe filter and used for further studies.

### 2.4. Liposome and Micelle Size, Polydispersity Index, and Zeta Potential Measurement

The size of the obtained formulations (MDD), polydispersity index (PDI), and zeta potential (ZP) were measured using Zetasizer Nano ZS (Malvern Instruments, Malvern, UK) for micelles, and for liposomes Zetasizer Ultra (Malvern Panaalytical, Malvern, UK). These systems are based on dynamic light scattering (DLS technology, Ottawa, ON, Canada). Measurements were performed in triplicate. To achieve optimal particle concentration, 10.0 µL of liposomes or 100.0 µL of micelles suspension was diluted with 10.0 mL of water for injection. Zeta potential measurements were conducted using U-shaped cuvettes fitted with gold electrodes. Particle size distributions were determined by dynamic light scattering (DLS) using disposable square polystyrene cuvettes.

### 2.5. Chromatographic Conditions

The NG and FCur concentrations in NFs were determined using the HPLC method. The developed method was carried out in gradient conditions with DAD detection on analytical apparatus comprising Agilent 1260 Infinity II LC System (Agilent Technologies, Bolinem, Germany) equipped with a quaternary pump (model G7111B) and degasser, a vial sampler (model G7129A) set at 15 °C, multicolumn thermostat (model G7116A) set at 25 °C, and diode array (DAD WR, model G7115A) detector. Gradient elution was based on a mobile phase consisting of two solvents: A–acetic acid in water (0.4% CH_3_COOH), and solvent B–acetonitrile with a constant flow rate of 1 mL/min throughout run (21 min). The volume ratio (*v*/*v* in %) of the mobile phase (A:B) was initially 50/50 at 0 min to 4 min, changed to 35/65 at 4 min to 5 min, then was 35/65 to 16 min, and returned to the initial ratio of 50/50 at 17 min and remained so until the end of the analysis at 21 min. The reverse phase column used as the stationary phase (C-18(2) 100 Å Luna^®^, 150 × 4.6 mm ID, 5 µm, Phenomenex, Torrance, CA, USA), stored at 25.0 ± 0.8 °C. Chromatograms were recorded at the maximum absorption of the substances (480 nm for FCur, and 289 nm for NG).

### 2.6. HPLC Method Validation

In order to confirm the suitability of the developed gradient LC method for the simultaneous determination of compound FCur and NG, the method was validated with respect to selectivity, linearity, range, sensitivity, precision, and accuracy (expressed as recovery, %) according to the ICH guidelines for analytical method validation (Q2(R2)) [38]. Stock solutions of FCur and NG (20 µg/mL) were prepared and further diluted appropriately to obtain calibration standards in the concentration ranges of 0.20–12.01 µg/mL for FCur and 0.14–6.81 µg/mL for NG. Precision and accuracy, both intra- and inter-day, were assessed using samples at concentrations of ~2.20, 4.20, and 8.40 µg/mL for FCur; and 1.40, 2.80, and 5.41 µg/mL for NG, prepared in six replicates, on two different days.

**Figure 2:**
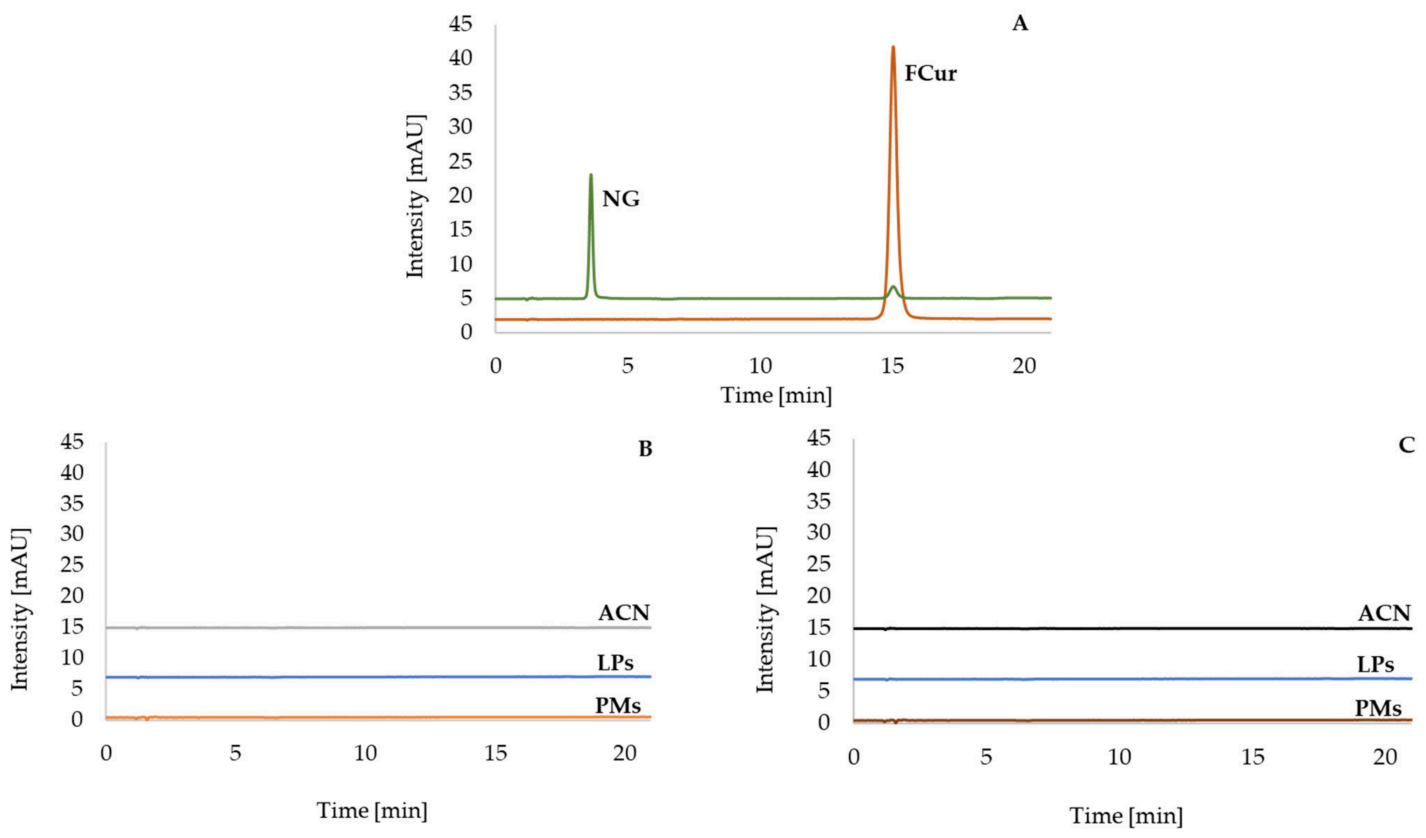
HPLC chromatograms of NG and FCur recorded at wavelengths corresponding to their maximum absorption peaks: 289 nm for NG and 480 nm for FCur (**A**). Chromatograms of acetonitrile (ACN), Soluplus^®^ empty micelles (PMs) solutions in ACN, and empty liposomes (LPs) in an ACN:EtOH (1:1) mixture at 480 nm (**B**) and 289 nm (**C**).

During validation, it was established that the gradient HPLC method for the simultaneous determination of FCur and NG was selective, linear, accurate, and precise. The selectivity of the developed method was evaluated by verifying the chromatograms of FCur and NG, blank samples of empty liposomes and micelles, and the solvents (ACN). Examples of chromatograms are presented in Figure 2.

The calibration curves for both compounds showed linearity over the concentration ranges of 0.20–12.01 µg/mL (*n* = 6) for FCur and 0.14–6.81 µg/mL (*n* = 8) for NG, with a correlation coefficient r > 0.999. The relationships between P and c (P = f(c)) were described by the equations y = ax since the regression coefficients *b* were found to be statistically insignificant according to Student’s *t*-test (*t*_b_ < *t*_0.05_, (*n* − 2)). The LOD and LOQ values were 0.050 µg/mL and 0.151 µg/mL for FCur, and 0.045 µg/mL and 0.137 µg/mL for NG, respectively. The method exhibited satisfactory precision, both intra-day and inter-day, as indicated by the percentage relative standard deviation (RSD%) for FCur (between 0.04 and 1.3%) and for NG (between 0.31 and 0.51%). The mean percentage recoveries of 99.82–101.64% for NG and 98.67–99.96% for FCur indicate the method’s high accuracy.

### 2.7. Encapsulation Efficiency (EE%)

The encapsulation efficiency (EE) of FCur, NG, and their mixtures were calculated according to Formula (1):

EE% = (C_en_ × 100)/C_in_ (1)

where C_en_—is the actual amount of the substance in the formulation measured using the HPLC method, C_in_—is the initial amount of the substance used for the preparation of the liposomes or micelles. EE was evaluated by HPLC after disruption of the nanocarrier systems. Micellar samples were treated with acetonitrile, while liposomal samples were mixed with an acetonitrile/ethanol mixture (1:1, *v*/*v*). The prepared samples were analyzed by HPLC, with a 10 μL injection volume.

### 2.8. NMR Analysis

^1^H NMR experiments were performed using a pulse spectrometer, operating at 30.2 MHz for liposomes and micelles and at 25 MHz for lyophilized micelles. The proton spin-lattice relaxation time (*T*_1_) in the laboratory frame was determined using a standard saturation pulse sequence $\left(n\times 90_{x}^{0}-t-90_{x}^{0}\right)$ and by fitting the equation:

(2) $$M\left(t\right)=M_{0}\left(1-exp\left(-\frac{t}{T_{1}}\right)\right),$$

to the experimental data, where *M*(*t*) is the magnetization at time *t*, *M*_0_ denotes the equilibrium magnetization, and *T*_1_ represents the spin–lattice relaxation time. All measurements were performed with an estimated uncertainty of a few percent. The spin-spin relaxation time *T*_2_ in the laboratory frame was measured using a standard spin-echo pulse sequence $\left(90_{x}^{0}-t-180_{x}^{0}\right)$. For all experiments, the decay curves were fitted using a combination of Gaussian and Lorentzian functions:

(3) $$M\left(t\right)=M_{0G}exp\left(-{\left(\frac{t}{T_{2G}}\right)}^{2}\right)+M_{0L}exp\left(-\frac{t}{T_{2L}}\right).$$

The *T*_1_ and *T*_2_ relaxation times of the liposomal and polymeric micellar systems were measured at room temperature, whereas *T*_1_ relaxation measurements of the lyophilized micellar systems were performed over the temperature range of 80–286 K.

To investigate the molecular dynamics of the lyophilized systems (FCur-PMs, NG-PMs, MIX-PMs, PMs), it was assumed that the dominant spin-lattice relaxation mechanism originates from dipole–dipole interactions described by the Bloembergen-Purcell-Pound (BPP) theory, modulated by thermally activated molecular motions [39]. The experimental temperature dependence of the relaxation times was analyzed using Equation (4) derived from the theory of dipolar relaxation, taking into account all contributing relaxation processes.

The experimental data were fitted numerically using the following equation:

(4) $$\frac{1}{T_{1}}=\frac{2}{3}\gamma^{2}\sum ∆M_{2k}\left(\frac{\tau_{ck}}{1+\omega_{0}^{2}\tau_{ck}^{2}}+\frac{4\tau_{ck}}{1+{4\omega }_{0}^{2}\tau_{ck}^{2}}\right),$$

where *γ* is the proton gyromagnetic ratio, Δ*M*_2*k*_ denotes the reduction in the second moment of the ^1^H NMR line associated with the (k)-th molecular motion, and $\tau_{ck}$ is the correlation time corresponding to the respective motional process. Assuming that the molecular motions are thermally activated, the correlation times were described using the Arrhenius Equation (5):

(5) $$\tau_{ck}=\tau_{0}exp\left(\frac{E_{ak}}{RT}\right),$$

where *τ*_0_ is the pre-exponential factor, *E_ak_* presents the activation energy of molecular motion, and *R* is the gas constant.

### 2.9. Biological Activity Assessment (MTT Test)

The effects of FCur and NG, and their NFs, on cell viability were assessed using the MTT assay. The 5637 bladder cell line, as a primary bladder tumor, was cultured in RPMI-1640 Medium, whereas the LNCaP cell line, as a metastatic prostate cancer, and the MRC-5 non-cancerous human fibroblast cell line were cultured in Dulbecco’s Modified Eagle’s Medium (DMEM). All media contained 10% fetal bovine serum (EURx) and 1% antibiotic solution, and cells were cultured at 37 °C, in a humidified 5% CO_2_ atmosphere. The 5637, LNCaP, and MRC-5 cells were seeded (10,000 per well) in 96–well plates. After 24 h of incubation, the cells were treated with compounds or their formulations in concentrations of 1–50 µM and incubated for an additional 24 h, 48 h, and 72 h. Cells were then washed twice with phosphate-buffered saline (PBS) and incubated again for 4 h in a medium supplemented with 0.5 mg/mL 3-(4,5-dimethylthiazol-2-yl)-2,5-diphenyl-2H-tetrazolium bromide (MTT). The formed formazan crystals were dissolved in acidic isopropanol. The absorbance was measured at 570 and 690 nm. The experiment was repeated three times. IC_50_ values were calculated from normalized concentration–response data using nonlinear regression with a four-parameter logistic dose–response model. The untreated control was defined as 100% cell viability, and compound concentrations were analyzed on a logarithmic scale. When the cell viability did not decrease below 50% within the tested concentration range, the IC_50_ value was reported as >50 µM. 

### 2.10. Statistical Analysis

All quantitative data are presented as mean ± SEM. For the MTT assay, *n* = 3 refers to three independent biological experiments conducted on separate occasions. Technical replicates within each independent experiment were averaged before statistical analysis and were not treated as independent biological replicates. Cell viability values were normalized to untreated control cells, defined as 100% viability.

MTT viability data were analyzed separately for each cell line and each incubation time using one-way ANOVA at each concentration, followed by Dunnett’s post hoc multiple-comparison test versus LPs. Adjusted *p*-values were used to determine statistical significance. Differences were considered statistically significant at adjusted *p* < 0.05.

The MTT viability curves show the relationship between the initial input concentration used in formulation preparation (in logarithmic scale) and the corresponding cell viability response. The IC_50_ values were corrected for the experimentally determined encapsulation efficiency, based on HPLC-measured concentrations of the active compounds in the final formulation after disruption of the nanocarrier system. Therefore, the reported IC_50_ values reflect the concentrations of the active compounds added to cells. For mixed formulations, the reported concentrations correspond to the sum of both active compounds.

## 3. Results and Discussion

Poor aqueous solubility remains a major limitation affecting the therapeutic application of many bioactive compounds, such as naringenin, curcumin, and its derivatives. One way to improve the solubility, physicochemical stability, and bioavailability of these compounds, and to modulate their pharmacological activity, is to encapsulate them within various nanoscale delivery systems [31]. Moreover, recently, increasing attention has been directed toward the co-encapsulation of naturally derived bioactive compounds within nanoscale carriers, which can enhance therapeutic efficacy compared to single-component formulations.

In this study, a fluorinated curcumin derivative, naringenin, and their mixture were encapsulated in polymeric and liposomal NFs. The obtained systems were characterised in aqueous dispersion for their physicochemical properties, including particle size, polydispersity index, zeta potential, and encapsulation efficiency. Then, in vitro cytotoxicity against cancer and normal cell lines was then evaluated. Additionally, nuclear magnetic resonance (NMR) relaxation studies were performed to investigate the molecular dynamics of both formulations in dispersion. Based on these results, the micellar systems were subjected to lyophilisation. After reconstitution, their physicochemical properties and molecular dynamics were evaluated by NMR to assess the effects of freeze-drying.

### 3.1. Compositions and Preparation of Liposomes and Micelles

In order to develop polymeric and liposomal NFs for FCur and NG using the thin-film hydration method, the composition and preparation conditions were optimized. In the first step for the liposome preparation study, various lipid compositions were tested. Preliminary experiments showed that formulations consisting solely of POPC were unable to effectively incorporate FCur and did not yield liposomal systems. Consequently, the cationic lipid DOTAP was introduced. Several POPC:DOTAP molar ratios (7:0.7, 8:0.5, 8:0.7, and 8:0.8) were evaluated. Among the tested formulations, the 8:0.8 ratio yielded the most favorable physicochemical properties and was therefore selected for further study. Next, the effect of the preparation conditions on the liposome properties was evaluated. Membrane extrusion yielded liposomes with a narrow particle size distribution (PDI < 0.3), but simultaneously led to a marked decrease in the encapsulation efficiency of FCur, which reached only about 7%. In contrast, sonication in an ultrasonic bath maintained a high encapsulation efficiency (>70%) despite a slightly broader particle size distribution (PDI > 0.3). Given the significant improvement in FCur encapsulation, sonication was selected as the preparation method for subsequent experiments.

For polymeric micelles, the formulations were prepared using different concentrations of Soluplus^®^ (105.0 mg/mL, 62.0 mg/mL, and 31.5 mg/mL), while maintaining a constant amount of encapsulated compounds. Micelles prepared using 105.0 mg/mL Soluplus^®^ exhibited immediate precipitation of FCur and NG upon hydration, whereas formulations containing 62.0 mg/mL of the polymer remained stable only for a limited time before precipitation occurred. In contrast, no visible precipitation was observed in systems prepared with 31.5 mg/mL Soluplus^®^, indicating that an optimal polymer-to-encapsulant ratio had been achieved. However, the particle size distribution parameters obtained under these conditions remained suboptimal. Consequently, the effect of mixing time on micelle formation was investigated, using mixing periods of 15, 20, and 30 min. Among the tested conditions, 30 min of mixing yielded the most favourable physicochemical properties of the NF, including the mean particle diameter and polydispersity index, and therefore, these were selected for further characterisation and biological evaluation. The physicochemical properties of formulations evaluated during the optimization process are presented in Supplementary Table S2.

Based on the optimization, it was determined that the production of NFs by the thin-film hydration method required sonication (9–12 min) for liposomes, and 30 min of shaking for micelles. Finally, the following formulation compositions were chosen: POPC:DOTAP in an 8:0.8 ratio for liposomes; 31.5 mg/mL Soluplus^®^ for polymeric micelles; and, for both NFs, an API concentration of 100 µM for a single NF (FCur or NG) or 50 µM of each compound for mixed formulations.

### 3.2. Physicochemical Characterisation of Liposomes and Micelles

Physicochemical analysis of nanocarriers provides important insight into their structural organisation and potential pharmaceutical applicability [40,41]. The physicochemical characterisation of the developed nanocarrier systems revealed substantial differences between polymeric micelles and liposomal formulations in terms of particle size, dispersity, and surface charge (Table 1).

**Figure 3:**
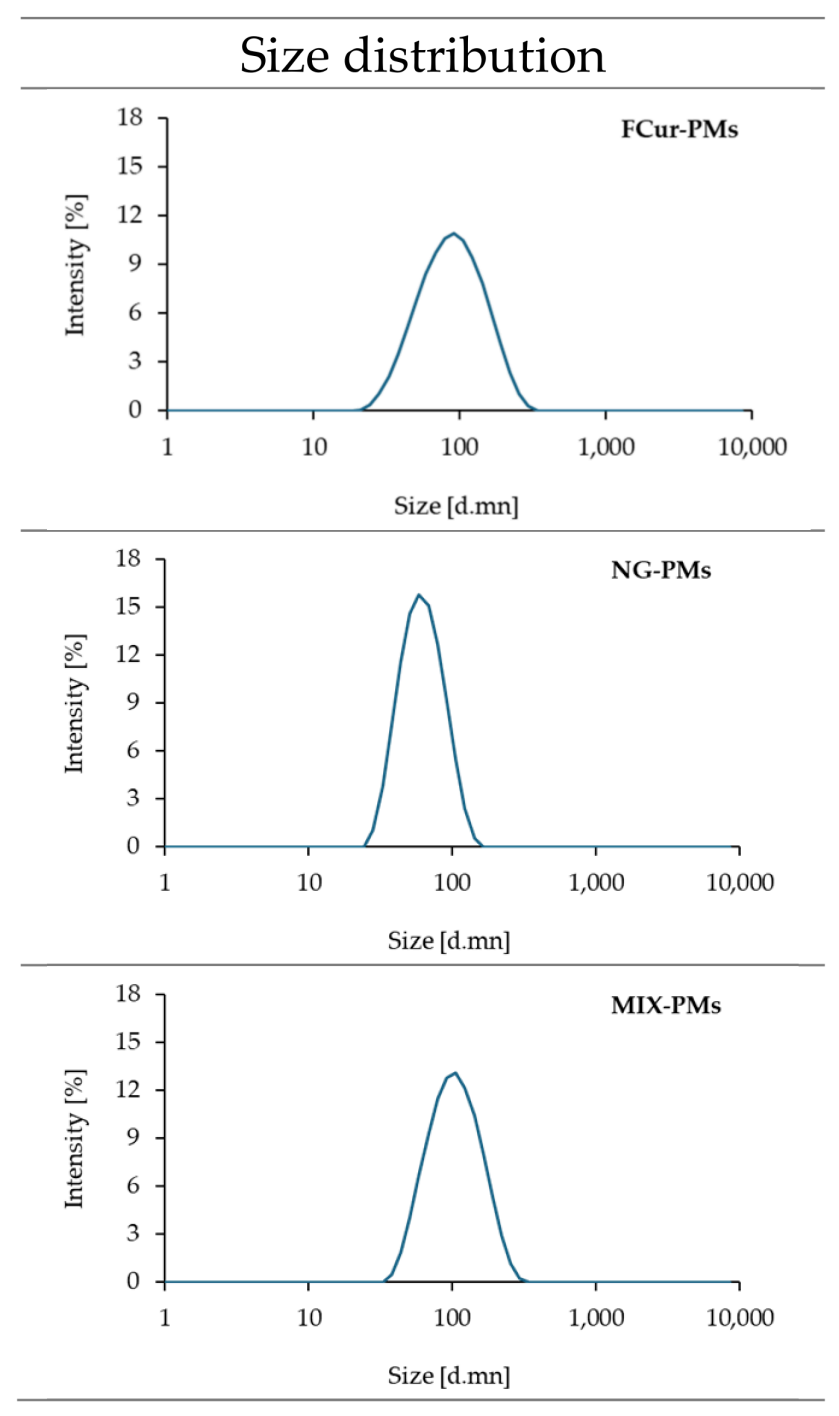
Particle size distribution of Soluplus^®^-based NFs determined by DLS. Data are presented as the mean of three measurements (*n* = 3).

The developed Soluplus^®^-based micelles formed relatively small and homogeneous nanoparticles with diameters below 100 nm and low PDI values (<0.20), indicating efficient self-assembly of the amphiphilic polymer matrix (Table 1, Figure 3). The absence of significant changes in particle size and size distribution following drug loading indicates that the incorporation of FCur and NG did not interfere with the self-assembly of Soluplus^®^ molecules. Similar physicochemical characteristics were reported by Wang et al. [42], and Ji et al. [43], who described curcumin-loaded self-assembled micelles with sizes below 100 nm and PDI in range 0.109–0.420.

**Figure 4:**
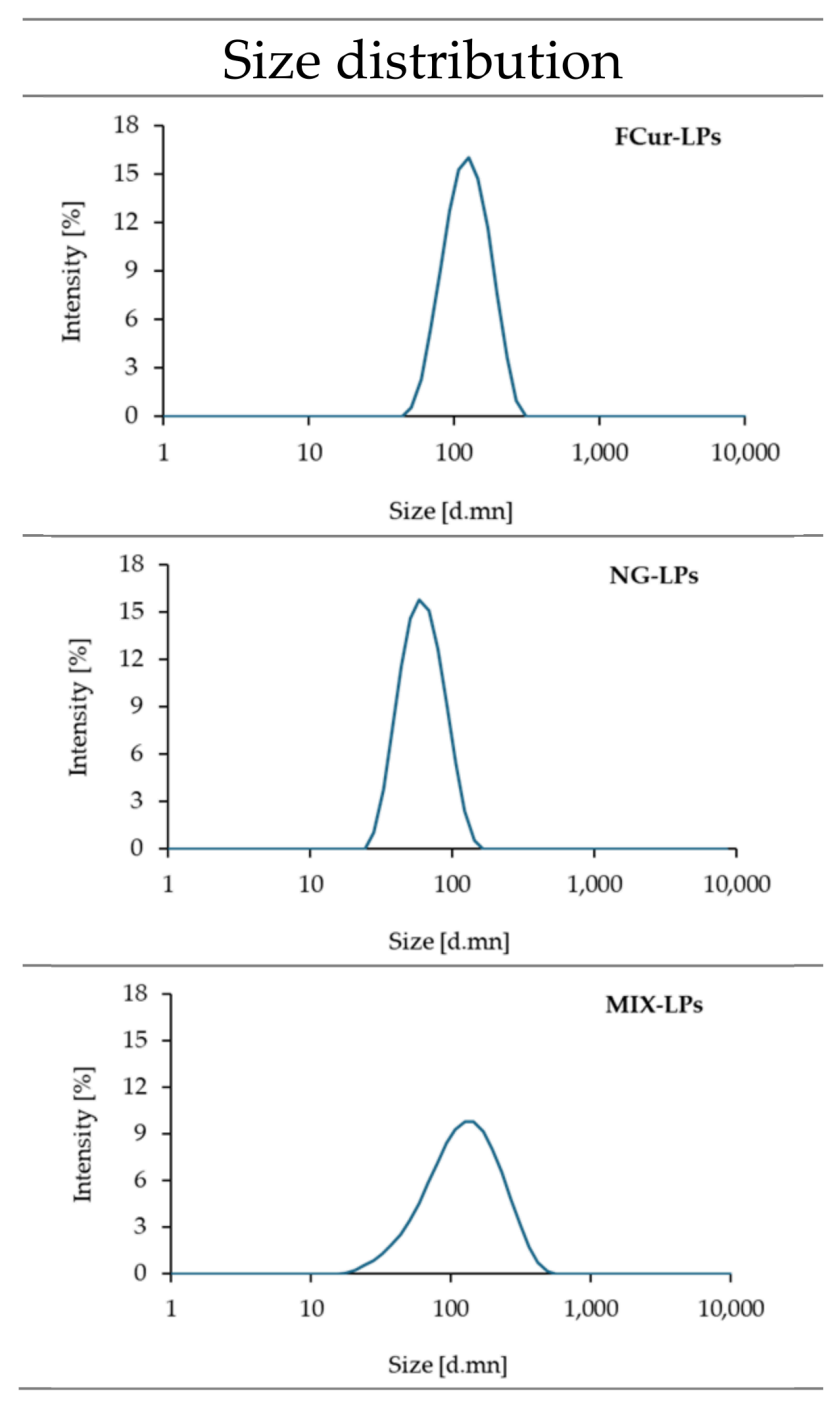
Particle size distribution of liposomal-based NFs determined by DLS. Data are presented as the mean of three measurements (*n* = 3).

In contrast to micelles, LPs exhibited significantly larger particle sizes (98–160 nm, *p* < 0.05), and broader size distributions (PDI values between 0.23 and 0.37, *p* < 0.05). This finding reflects a general structural feature of phospholipid bilayer systems (Table 1, Figure 4). In the context of lipid-based nanocarriers used for drug delivery, such as liposomes and nanoliposomes, a PDI value of ≤0.3 is commonly interpreted as indicative of an acceptable and relatively homogeneous phospholipid vesicle population. However, acceptable PDI values may vary depending on the intended route of administration, the complexity of the formulation, and its final dosage form [44]. For example, an optimized epirubicin-loaded NF developed for intravesical bladder cancer therapy exhibited a PDI of 0.47 while maintaining enhanced urothelial penetration and anticancer activity. This finding indicates that moderate particle size heterogeneity does not necessarily compromise the performance of locally administered nanocarriers [45]. Likewise, cationic liposomal formulations containing dimethyldioctadecylammonium and D-(+)-trehalose 6,6′-dibehenate have been reported with PDI values between 0.3 and 0.5 while maintaining other physicochemical properties and biological activity [46]. Although the most recent FDA “Guidance for Industry” on liposome drug products recognizes particle size and size distribution as critical quality attributes (CQAs) and key parameters for stability evaluation, it does not provide explicit acceptance limits for PDI [47].

**Table 1:** Particle size (MDD), polydispersity index (PDI), and zeta potential (ZP) of Soluplus^®^-based micelles (PMs), and liposomes (LPs).

Sample	MDD ± SD [nm]	PDI ± SD	ZP ± SD [mV]
Micelles			
FCur-PMs	79.00 ± 12.93	0.163 ± 0.01	−3.90 ± 1.75
NG-PMs	92.08 ± 28.55	0.102 ± 0.05	−2.05 ± 0.13
MIX-PMs	78.76 ± 8.02	0.199 ± 0.03	−3.27 ± 2.19
PMs	92.63 ± 27.22	0.168 ± 0.02	−4.31 ± 2.39
Liposomes			
FCur-LPs	138.90 ± 18.95	0.328 ± 0.03	+44.87 ± 0.55
NG-LPs	97.96 ± 2.81	0.227 ± 0.01	+41.69 ± 1.41
MIX-LPs	115.15 ± 16.33	0.250 ± 0.01	+42.59 ± 2.11
LPs	160.60 ± 3.51	0.369 ± 0.04	+48.65 ± 8.70

Moreover, in liposomal formulations, incorporation of the investigated compounds resulted in a reduction in particle size. While FCur-LPs and MIX-LPs exhibited only a moderate decrease in diameter compared with empty liposomes, encapsulation of NG led to a pronounced size reduction (from 160 nm to 98 nm). This observation suggests that the effect of compound incorporation on liposome size depends on the specific interactions between the encapsulated molecule and the phospholipid bilayer. Previous studies have shown that naringenin can interact with phospholipid headgroups through hydrogen bonding, leading to enhanced lipid packing and formation of smaller vesicles. The pronounced size reduction observed for NG-LPs is therefore consistent with these reports [48,49]. Curcumin derivatives, on the other hand, are known to localise within the hydrophobic acyl-chain region of lipid bilayers and modulate membrane organization [50]. However, the fluorinated curcumin derivative investigated in the present study produced only a moderate decrease in vesicle size. This finding suggests a different mode or extent of interaction with the lipid membrane.

The zeta potential values of the micellar systems remained, on average, slightly negative, at approximately −3.4 mV, and remained comparable for all micellar systems, indicating that electrostatic stabilisation was not the dominant mechanism responsible for colloidal stability. Instead, micelle stabilisation is most likely due to steric effects from the hydrophilic polymer chains surrounding the micelle core. Such behaviour is characteristic of nonionic polymer micelles and has been previously described for Soluplus^®^-based nanosystems [51]. The near-neutral surface charge may also contribute to the lower cytotoxicity of micellar formulations compared with cationic liposomes by reducing non-specific interactions with cellular membranes [52].

In contrary to micelles dispersions obtained, all liposomes exhibited strongly positive zeta potential, exceeding +48 mV, which should ensure the electrostatic stabilization of lipid dispersions [40,41]. The inclusion of active compounds slightly reduced the zeta potential relative to empty liposomes. This phenomenon is commonly observed in liposomal formulations due to the dynamic nature of phospholipid bilayers and the coexistence of vesicle populations differing in size [53].

### 3.3. Encapsulation Efficiency

Encapsulation studies demonstrated high encapsulation efficiency for both Soluplus^®^-based micelles and liposomal systems for the active compounds tested (Table 2). Depending on the formulation type, the encapsulation efficiency of FCur ranged from 72% to 80%. In contrast, NG showed significantly higher encapsulation efficiencies, consistently exceeding 95% (*p* < 0.05). This finding suggests more favourable interactions of NG with both polymeric and lipid carrier matrices. Complete encapsulation of active compounds is rarely achieved in practice, since the loading process depends on multiple factors, including the drug’s physicochemical properties, carrier composition, preparation method, and drug-carrier interactions [54]. Consequently, encapsulation efficiencies below 100% are commonly reported for both liposomal and polymeric micellar systems and do not necessarily indicate poor formulation performance [55,56,57]. The differences of FCur and NG encapsulation may stem from the distinct physicochemical properties of the compounds used, including their hydrophobicity and affinity for the nanocarrier. In silico analysis revealed higher lipophilicity of the FCur (logP_calc_. ≈ 5.1; calculated by SwissADME) compared to NG (logP_exp_ = 2.6 [58], logP_calc_. ≈ 1.9 or 2.4; calculated by SwissADME or XLogP3 3.0 [59], respectively). However, interactions with the lipid bilayer may also depend on other structural features, including the ability to form hydrogen bonds and the arrangement of functional groups within the molecule.

### 3.4. Biological In Vitro Activity

To evaluate the therapeutic potential of the nanoparticle-based delivery systems, the effects of free FCur, NG, and their respective NFs were investigated on the viability of bladder cancer (5637), prostate cancer (LNCaP), and non-cancerous fibroblast (MRC-5) cells.

**Figure 5:**
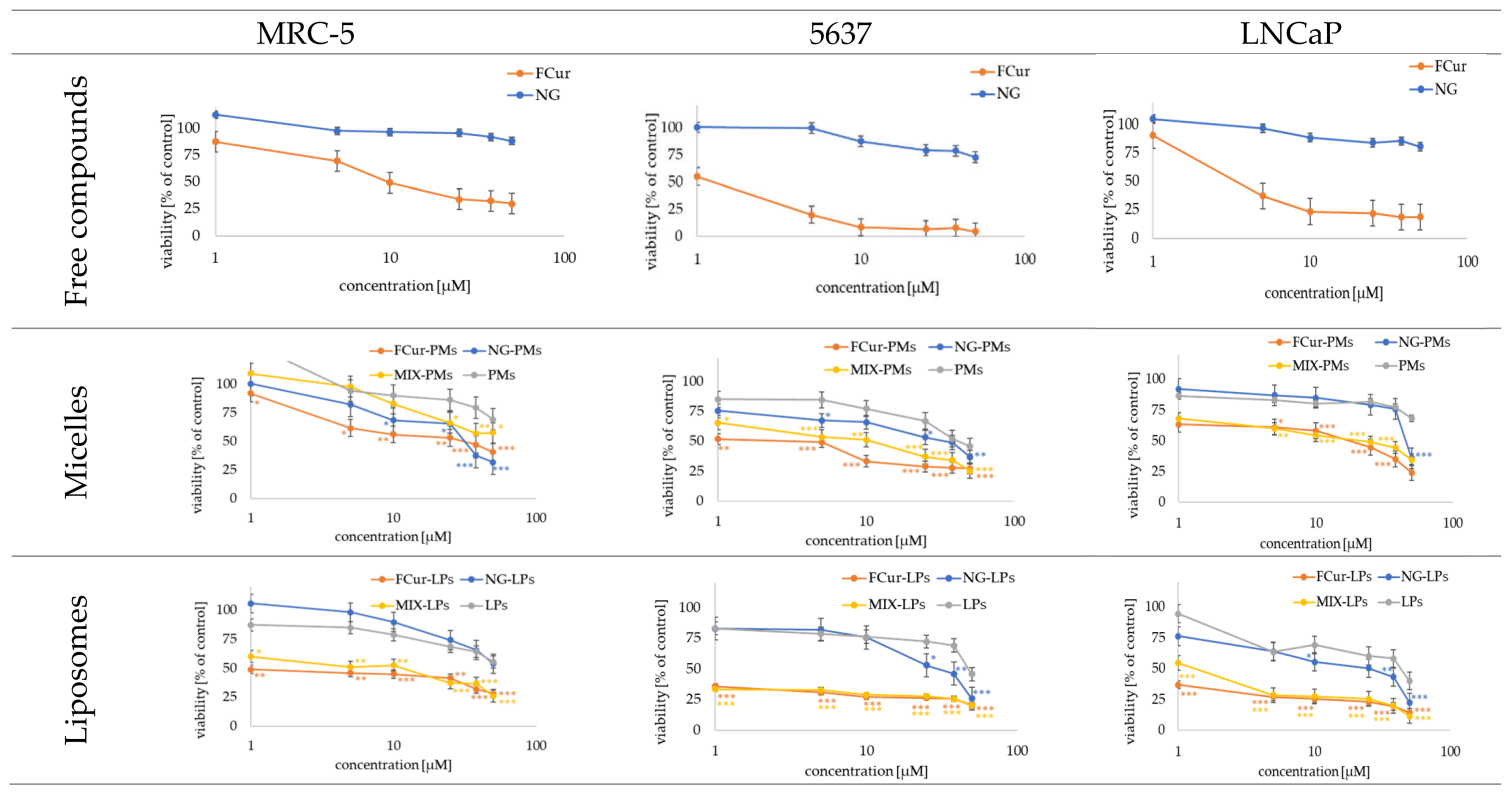
MTT viability curves for MRC-5 fibroblast cells, 5637 bladder cancer cells, and LNCaP prostate cancer cells after 24 h incubation with free compounds and their nanoformulations. Cell viability values were normalized to untreated control cells, defined as 100% viability. The data are shown as the mean ± SEM of three independent replicates. Statistical significance was assessed using Dunnett’s post hoc multiple-comparison test versus LPs:* *p* < 0.05; ** *p* < 0.01; and *** *p* < 0.001).

**Figure 6:**
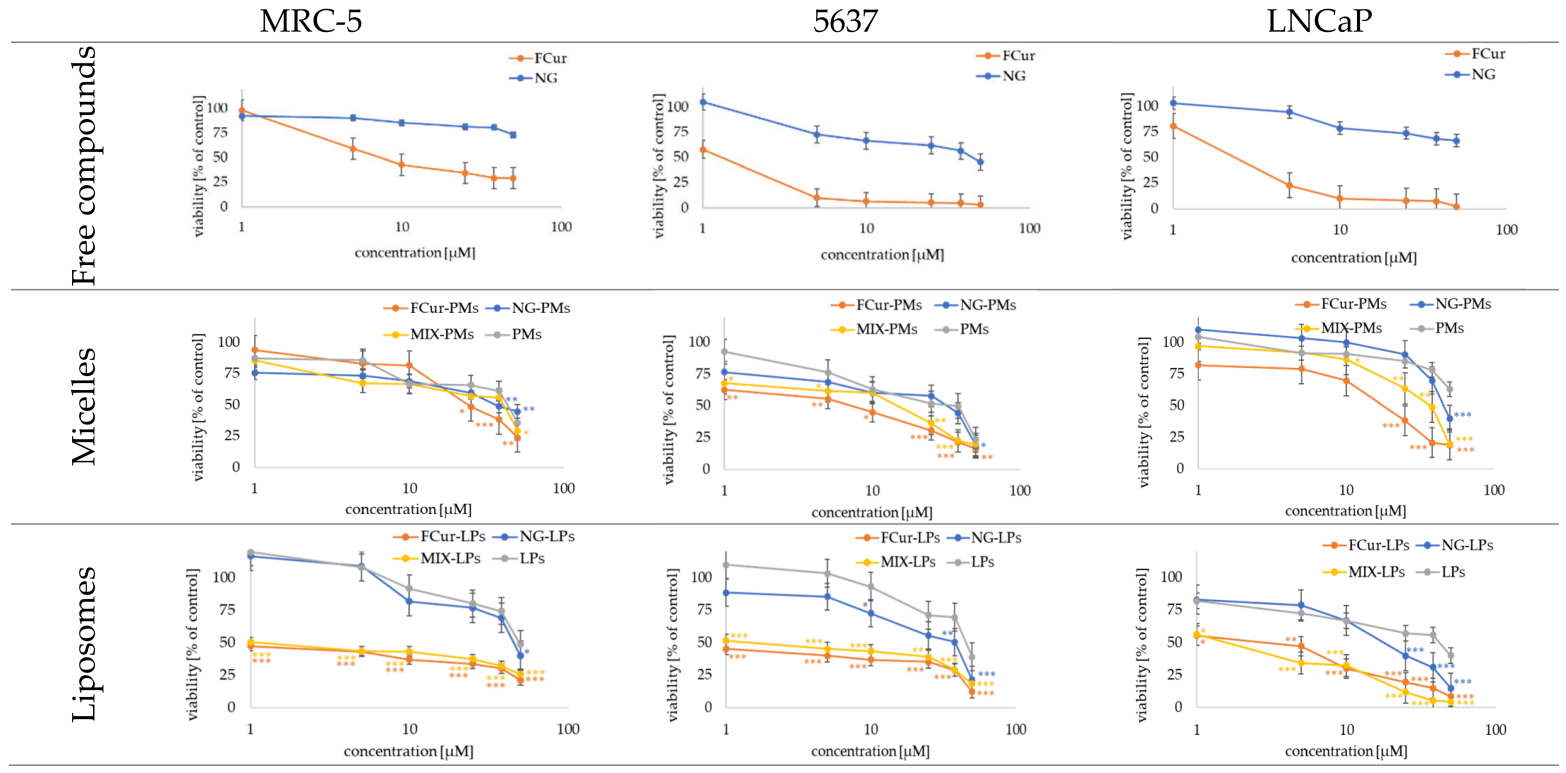
MTT viability curves for MRC-5 fibroblast cells, 5637 bladder cancer cells, and LNCaP prostate cancer cells after 48 h incubation with free compounds and their nanoformulations (NFs). Cell viability values were normalized to untreated control cells, defined as 100% viability. The data are shown as the mean ± SEM of three independent replicates. Statistical significance was assessed using Dunnett’s post hoc multiple-comparison test versus the LPs: * *p* < 0.05; ** *p* < 0.01; and *** *p* < 0.001).

**Figure 7:**
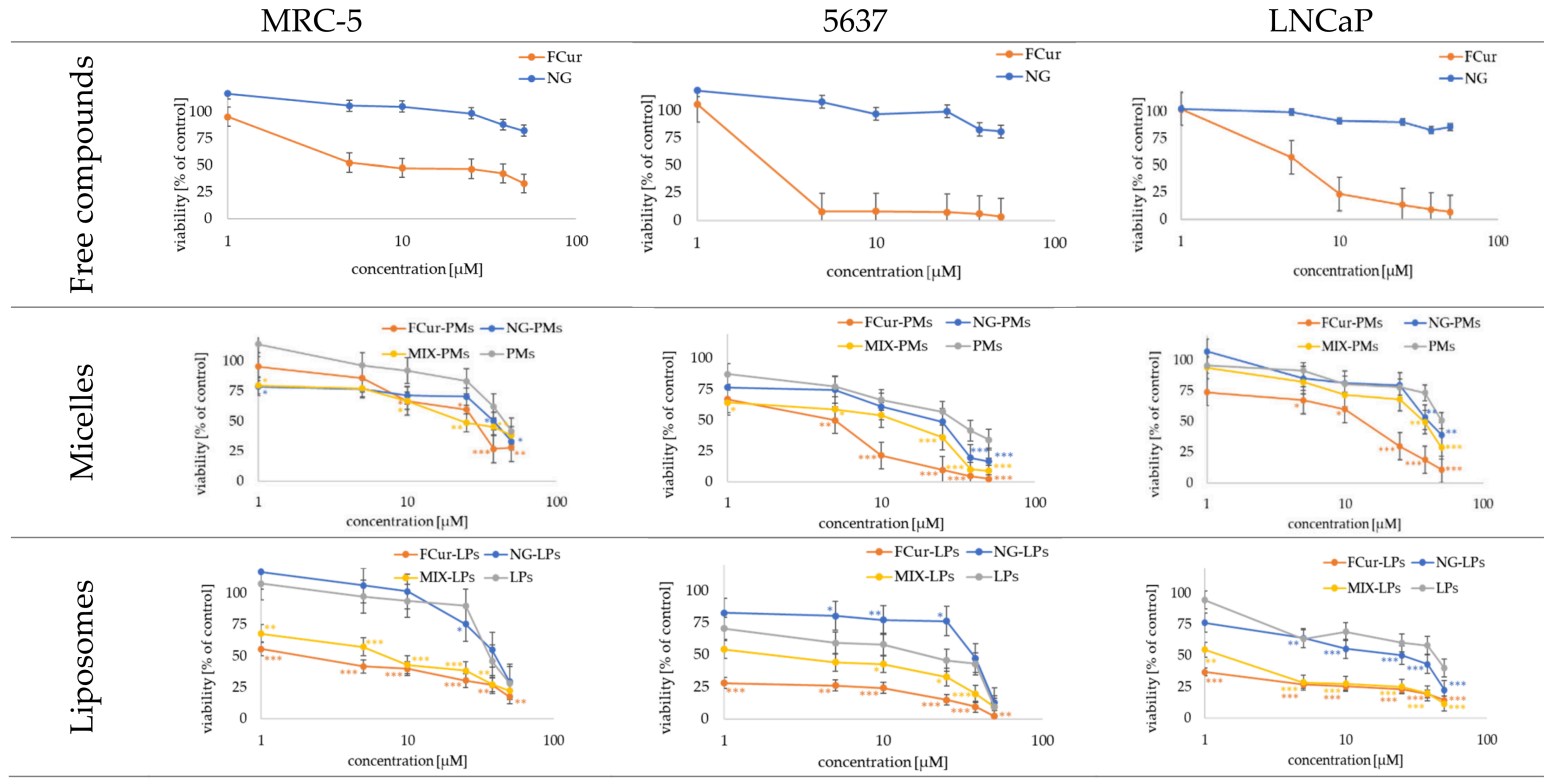
MTT viability curves for MRC-5 fibroblast cells, 5637 bladder cancer cells, and LNCaP prostate cancer cells after 72 h incubation with free compounds and their nanoformulations (NFs). Cell viability values were normalized to untreated control cells, defined as 100% viability. The data are shown as the mean ± SEM of three independent replicates. Statistical significance was assessed using Dunnett’s post hoc multiple-comparison test versus the LPs: * *p* < 0.05; ** *p* < 0.01; and *** *p* < 0.001).

MTT assays performed in the concentration range of 1–50 μM revealed distinct cytotoxicity profiles among the tested compounds and formulations (Table 3, Figure 5, Figure 6 and Figure 7). FCur exhibited significantly higher cytotoxic activity than NG in all tested cell lines. In both normal and cancer cell models, FCur markedly reduced cell viability at low micromolar concentrations (IC_50_ = 1.5–9.5 μM). These findings are consistent with previous reports describing the anticancer activity of curcumin and its synthetic derivatives in prostate and bladder cancer models [10,12,15]. NG displayed only limited activity, with IC_50_ values exceeding 50 μM, in both cancer cells and normal MRC-5 fibroblasts. This observation partially agrees with previous studies indicating that high NG concentrations are required to reduce prostate cancer cell viability. IC_50_ values reported in the literature for free NG vary considerably; for instance, Rathi et al. obtained 17.5 μM after 48 h incubation in LNCaP cells [7], whereas Gao et al. demonstrated values exceeding 300 μM after 24 h [60].

Encapsulation markedly influenced the biological effect of both compounds, although the magnitude of this effect depended on the carrier system.

Encapsulation in liposomes substantially enhanced the biological activity of both compounds. FCur-LPs exhibited markedly lower IC_50_ values than the free compound and the corresponding micellar formulations. FCur-LPs consistently displayed IC_50_ values between <1 and 3 μM, whereas MIX-LPs showed IC_50_ values ranging from <1 to 5.7 μM across all tested cell lines and incubation times. Liposomal formulations produced a pronounced concentration-dependent reduction in cell viability. These results are consistent with previously reported improvements in the anticancer activity of liposomal curcumin formulations in 5637 and LNCaP cells. Together, these findings support the concept that nanoencapsulation enhances the intracellular delivery and biological efficacy of hydrophobic polyphenols [60]. Encapsulation also improved the biological activity of NG, although to a lesser extent than that observed for FCur. While free NG displayed little or no anticancer activity under the tested conditions (IC_50_ > 50 μM), NG-loaded liposomes showed measurable biological activity, particularly against LNCaP cells, where an IC_50_ value of 25 μM was observed after 24 h of incubation. Overall, NG-LPs displayed IC_50_ values ranging from 9.9 to 38 μM across cancer cell lines, indicating partial improvement in biological activity upon liposomal encapsulation.

It is worth noting that although liposomal encapsulation enhanced cytotoxic activity, the results do not indicate that the investigated liposomal systems exhibit a consistent cancer-selective profile; rather, they reflect an overall increase in cytotoxicity under the investigated conditions. These findings highlight the importance of balancing efficacy and safety in the evaluation of nanocarrier-based formulations.

Polymeric micelles altered the biological profiles of both compounds but exhibited lower cytotoxic activity compared with liposomal systems. Nevertheless, FCur-loaded micelles retained substantial cytotoxicity against both cancer cell models, particularly in 5637 cells, where IC_50_ values ranged from 3 to 5 μM. The MIX-PMs formulation exhibited intermediate activity relative to the corresponding single-drug systems; with IC_50_ values of approximately 10–13 μM in 5637 cells. Notably, the viability curves obtained for MIX-PMs generally followed those of FCur-PMs rather than NG-PMs. Moreover, FCur-PMs and MIX-PMs induced a more pronounced concentration-dependent reduction in cell viability than NG-PMs in all tested cell lines, indicating that FCur was the main contributor to the cytotoxic response of the mixed formulations.

Neither liposomal nor polymeric mixed systems provided a clear advantage over the corresponding single FCur-loaded formulations. These findings suggest that the biological activity of the mixed formulations was primarily determined by FCur, while the contribution of NG to the overall cytotoxic effect remained limited within the tested concentration range. Nevertheless, the successful incorporation of both compounds into the same nanocarrier systems confirmed the feasibility of developing stable mixed formulations without compromising their physicochemical properties.

In the literature, Soluplus^®^-based micellar systems loaded with curcumin have generally been shown to enhance the biological activity of the encapsulated drug compared with the free compound. For example, Ji et al. reported a reduction in IC_50_ values from 54.5 μM for free curcumin to 28.6 μM and 10.3 μM for curcumin-loaded polymeric micelles and TPGS/Soluplus^®^ mixed micelles, respectively, in MCF-7 cells [43]. For comparison, free curcumin exhibited an IC_50_ value of 12.7 μM in 5637 bladder cancer cells [61]. In contrast, in the our study no clear enhancement of cytotoxicity was observed upon encapsulation of FCur in polymeric micelles, as free FCur displayed slightly lower IC_50_ values (1.5–4 μM) than FCur-PMs (3–5 μM). However, both free FCur and FCur-PMs demonstrated higher activity than values reported for free curcumin in 5637 cells, indicating that structural modification of curcumin (fluorination) may contribute to its improved anticancer potency.

It is worth noting that FCur-loaded polymeric micelles exhibited stronger effects against cancer cells than against normal MRC-5 fibroblasts, which represents a potential advantage of the developed micellar system over liposomal formulations, and free compound, despite its lower overall cytotoxicity.

The differences in cytotoxic activity between liposomal and polymeric systems may be partly associated with their surface charge characteristics. The liposomes developed in this study exhibited a strongly positive zeta potential (approximately +45 mV) arising from the presence of DOTAP, which may facilitate electrostatic interactions with negatively charged cell membranes. However, the positive surface charge of DOTAP-containing liposomes may also contribute to nonspecific cytotoxic effects by altering membrane integrity and modulating cellular responses [62,63].

The incorporation of POPC into DOTAP-based LPs may reduce the surface charge density and attenuate excessive electrostatic interactions with cell membranes, thereby improving the biocompatibility of the formulation while maintaining its drug-delivery capacity [64]. Indeed, Piwowarczyk et al. [61] demonstrated that POPC liposomes containing higher DOTAP proportions (9:1 and 8:2) exhibited cytotoxicity against LNCaP cells. Consequently, in the present study, a formulation containing a substantially lower proportion of DOTAP (POPC:DOTAP, 8:0.8) was selected to reduce the likelihood of carrier-associated cytotoxicity while preserving the advantages of a positively charged surface. The enhanced cytotoxicity of the liposomal formulations is most likely the result of multiple contributing factors, including improved intracellular delivery of the encapsulated compounds, electrostatic interactions between the positively charged carrier and the cell membrane, and the intrinsic biological activity of the cationic lipid component [62,65,66].

In contrast, Soluplus^®^-based micelles are based on a non-ionic amphiphilic copolymer and displayed near-neutral zeta potential values, suggesting limited contribution of electrostatic interactions.

Consistent with this interpretation, the empty LPs evaluated in the present study produced measurable reductions in cell viability, although their cytotoxic effects were markedly weaker than those of the corresponding drug-loaded formulations. In contrast, empty PMs exhibited only minimal cytotoxicity. These findings indicate that the enhanced biological activity of the drug-loaded formulations primarily reflects the pharmacological activity of the encapsulated compounds, while the cationic liposomal carrier also contributes to the overall biological response.

Despite similar encapsulation efficiencies of FCur and NG in both liposomal and micellar systems, significant differences in biological activity were observed. Such differences are likely influenced by multiple factors, including carrier composition, surface properties, drug release behaviour, and drug–carrier interactions.

**Table 3:** The IC_50_ values [µM] of free FCur, NG, and their formulations in polymeric micelles and liposomes. IC_50_ values were calculated by nonlinear dose–response fitting of normalized data and corrected for encapsulation efficiency based on HPLC-determined active compounds concentrations. Data are presented as mean ± SEM from at least three independent experiments.

Cell Line	Free Compounds		Micelles			Liposomes		
	FCur	NG	FCur-PMs	NG-PMs	MIX-PMs	FCur-LPs	NG-LPs	MIX-LPs
MRC5 24 h	9.5 ± 1.5	>50 ± 5.8	25.3 ± 6.4	31.5 ± 3.2	>50 ± 5.8	<1 ± 0.14	47.9 ± 5.5	4.4 ± 1.1
MRC5 48 h	7 ± 0.99	>50 ± 6.7	17.3 ± 4.3	34 ± 3.4	35.3 ± 4.9	<1 ± 0.15	44.6 ± 6.2	5.2 ± 2.1
MRC5 72 h	5.5 ± 0.67	>50 ± 6.6	21.2 ± 3.1	36.9 ± 4.6	29.1 ± 5.4	<1 ± 0.7	44.6 ± 4.9	5.7 ± 1.7
								
5637 24 h	1.5 ± 0.45	>50 ± 7.4	3 ± 1.1	26.2 ± 4.1	10.6 ± 2.8	<1 ± 0.13	29.7 ± 3.7	<1 ± 0.11
5637 48 h	2 ± 0.6	45 ± 6.3	5 ± 1.7	29.1 ± 2.8	10.2 ± 1.9	<1 ± 0.3	37.6 ± 3.8	1.9 ± 0.9
5637 72 h	4 ± 0.9	>50 ± 5.8	3.6 ± 1.8	28.1 ± 3.1	12.8 ± 2.5	<1 ± 0.25	35.3 ± 2.9	2 ± 1.5
								
LNCAP 24 h	4 ± 0.7	>50 ± 5.6	10.9 ± 2.3	44.6 ± 4.5	20.8 ± 2.4	<1 ± 0.4	24.8 ± 3.2	2.2 ± 0.11
LNCAP 48 h	3.5 ± 0.55	>50 ± 6.7	13 ± 1.9	44.6 ± 5.3	31.6 ± 2.8	<1 ± 0.23	19.3 ± 2.6	3 ± 0.2
LNCAP 72 h	6 ± 1.1	>50 ± 5.8	10.8 ± 2.5	38.3 ± 6.7	31.2 ± 4.1	<1 ± 0.3	9.9 ± 1.4	3 ± 0.9

Although liposomes and polymeric micelles are both widely used nanocarriers for drug delivery, they differ not only in their structural organization but also in their practical applications, engineering strategies, and stage of clinical translation. These differences influence the selection of an appropriate delivery system depending on the therapeutic objective and the physicochemical properties of the encapsulated drug. To provide a practical comparison of these nanocarriers, the key characteristics of both platforms are summarized in Table 4.

Liposomes are versatile systems capable of encapsulating both hydrophilic drugs within the aqueous core and hydrophobic drugs within the lipid bilayer. Their lipid composition enables direct interactions with cellular membranes, making them particularly suitable for targeted and intracellular drug delivery. Polymeric micelles, on the other hand, are primarily designed to encapsulate poorly water-soluble drugs. They improve the solubility, stability and pharmacokinetic properties of compounds with low bioavailability, while exhibiting minimal intrinsic biological activity. These differences are reflected in their engineering strategies, with liposomes typically optimized through surface functionalization and targeting approaches, whereas polymeric micelles are mainly engineered by tailoring polymer composition to maximize drug loading and formulation stability. The greater number of clinically approved liposomal formulations also demonstrates the higher translational maturity of this platform. Nevertheless, the clinical translation of both nanocarrier systems remains challenging. For polymeric micelles, systemic dilution may compromise structural stability and promote premature drug release, while the clinical use of some polymeric materials is further limited by insufficient safety characterization [70,80]. In contrast, the major translational challenges associated with liposomes are related to manufacturing complexity, quality control, storage stability, and scale-up [80]. Therefore, these nanocarriers should be regarded as complementary rather than competing technologies, and the choice between them should be guided by the therapeutic objective and the physicochemical properties of the encapsulated compound.

Although the literature analysis highlights differences between liposomes and micelles in terms of their applications, limitations, and challenges associated with their clinical translation, the properties of specific formulations are primarily determined by their composition and molecular organization. Therefore, following the characterization of the obtained systems in terms of their physicochemical and biological properties, as well as the comparison of both classes of nanocarriers based on literature data, the next step was to further characterize the investigated formulations at the molecular level. For this purpose, NMR relaxation studies were performed as a complementary technique to assess the molecular environment and mobility of the encapsulated compounds within the different nanocarriers.

### 3.5. ^1^H Nuclear Magnetic Resonance

In recent years, NMR methods have become increasingly important in the characterisation of modern drug carriers, including liposomes, polymeric nanoparticles, micelles, and amorphous solid dispersions. The technique enables investigation of drug-carrier interactions, molecular immobilisation, local molecular mobility, and the formation of heterogeneous domains within the carrier matrix. Such information is crucial for understanding the stability, encapsulation efficiency, drug release kinetics, and physicochemical properties of pharmaceutical formulations [83,84].

In this context, NMR relaxation studies provide detailed insight into the molecular dynamics of drug delivery systems and allow the evaluation of how active compounds influence local carrier mobility, phase heterogeneity, and intermolecular interactions [85,86,87]. These methods are therefore particularly valuable for characterising the molecular-level organisation and dynamic behaviour of the liposomal and polymer-based systems investigated in this work.

Since paramagnetic species may significantly affect NMR relaxation behaviour, EPR spectroscopy was additionally employed to verify their presence in the investigated formulations. No EPR signals were detected within the sensitivity range of the X-band spectrometer, indicating the absence of significant paramagnetic contributions to the observed relaxation process. Consequently, the measured NMR relaxation properties can be attributed to the intrinsic molecular dynamics and intermolecular interactions within the investigated nanocarrier systems.

#### 3.5.1. NMR Analysis of Liposomal Systems

**Figure 8:**
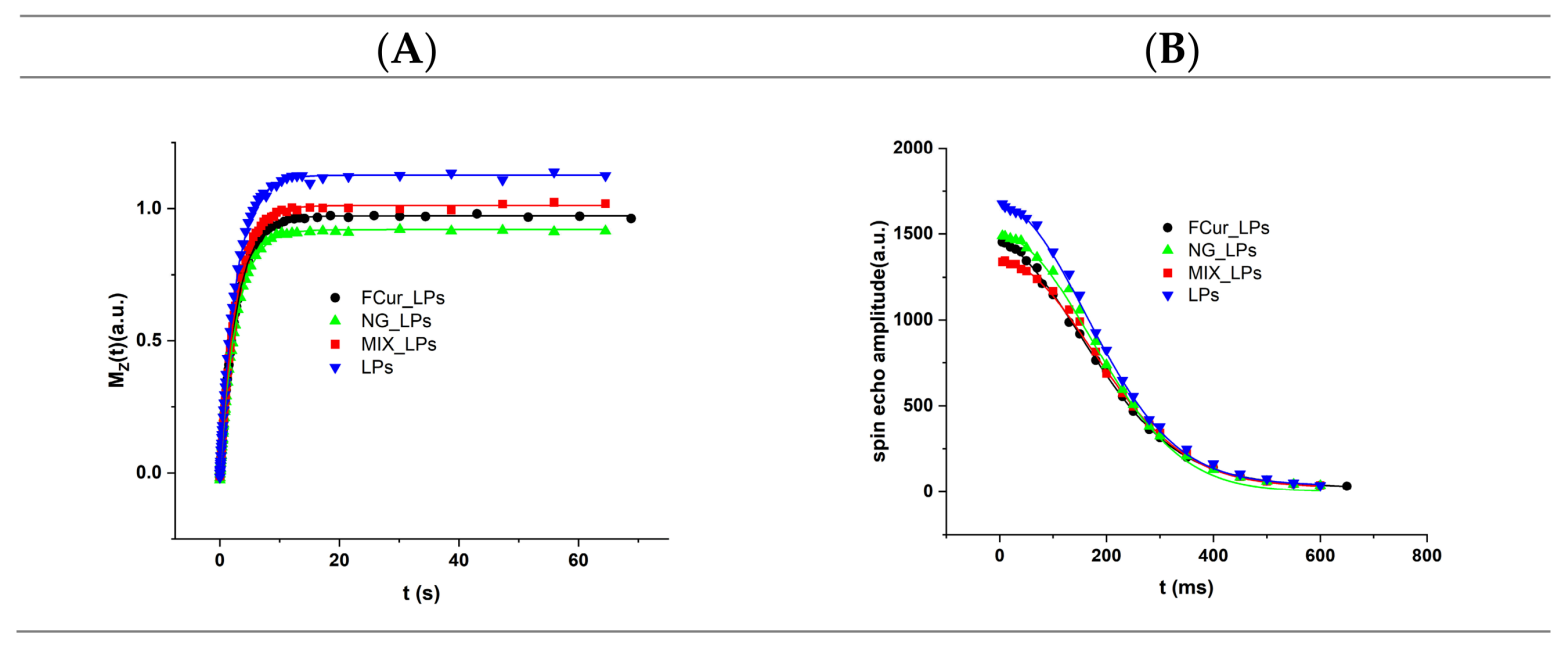
NMR relaxation analysis of liposomal formulations: (**A**): Time-dependent recovery of magnetisation (*T*_1_ relaxation) for all liposomal formulations; (**B**): Time-dependent decay of transverse magnetisation (*T*_2_ relaxation) for all liposomal formulations.

Representative magnetisation recovery curves and transverse relaxation decay profiles recorded for liposomal formulations are presented in Figure 8.

Only minor differences in the longitudinal relaxation behaviour were observed between the investigated formulations, indicating that incorporation of NG and FCur does not substantially alter the overall spin-lattice relaxation processes within the lipid bilayer. In contrast, the transverse relaxation decay profiles revealed heterogeneous relaxation behaviour. The data required a two-component model consisting of Gaussian (G-phase) and Lorentzian (L-phase) contributions, corresponding to more rigid and dynamically averaged membrane domains, respectively. The NG-LPs formulation exhibited the highest G-phase contribution, with an *M_o_G* contribution of 98%, suggesting restricted molecular mobility within the lipid bilayer. In contrast, incorporation of FCur increased the relative contribution of the Lorentzian component, reflecting enhanced motional averaging and increased local lipid mobility. These observations indicate that incorporation of FCur locally modifies membrane dynamics and intermolecular organisation within the liposomal structure.

The determined *T*_1_ and *T*_2_ relaxation parameters for all investigated liposomal systems are summarised in Table 5. Among the analyzed formulations, the mixed MIX-LPs system exhibited the highest transverse relaxation time for the Lorentzian component (*T*_2_*_L_* equal to 482 ms), indicating enhanced molecular mobility within dynamically averaged membrane domains. In contrast, incorporation of FCur alone reduced the *T*_2_*_L_* value to 252 ms, compared with 430 ms for the pure liposomal formulation, suggesting partial restriction of rotational mobility in the more mobile membrane regions. This effect may arise from specific interactions between the fluorinated curcumin derivative and phospholipid molecules, leading to local perturbations in membrane packing and dynamics.

The NG-LPs system retained relatively high lipid mobility (*T*_2*L*_ equal to 337 ms) despite the dominant contribution of the rigid Gaussian phase, indicating the coexistence of spatially restricted and dynamically mobile membrane domains. This behaviour may reflect compartmentalisation within the liposomal structure, in which naringenin helps maintain mobility within selected lipid environments despite the overall increase in membrane rigidity.

Since NMR measurements were performed within 48 h of sample preparation, the observed differences in relaxation times and phase contributions are primarily attributed to intrinsic modifications of bilayer dynamics induced by the encapsulated compounds rather than to liposome aggregation or long-term structural changes. Overall, the obtained results demonstrate that incorporation of NG and FCur significantly influences local molecular dynamics and lipid packing within liposomal systems while maintaining the structural integrity of the lipid bilayer.

#### 3.5.2. NMR Analysis of Micellar Systems

For all investigated micellar formulations, the presence of free radicals was evaluated using electron paramagnetic resonance (EPR) spectroscopy. No paramagnetic centers were detected within the sensitivity limits of the applied X-band spectrometer indicating the absence of significant paramagnetic contributions to the observed NMR relaxation behaviour.

**Figure 9:**
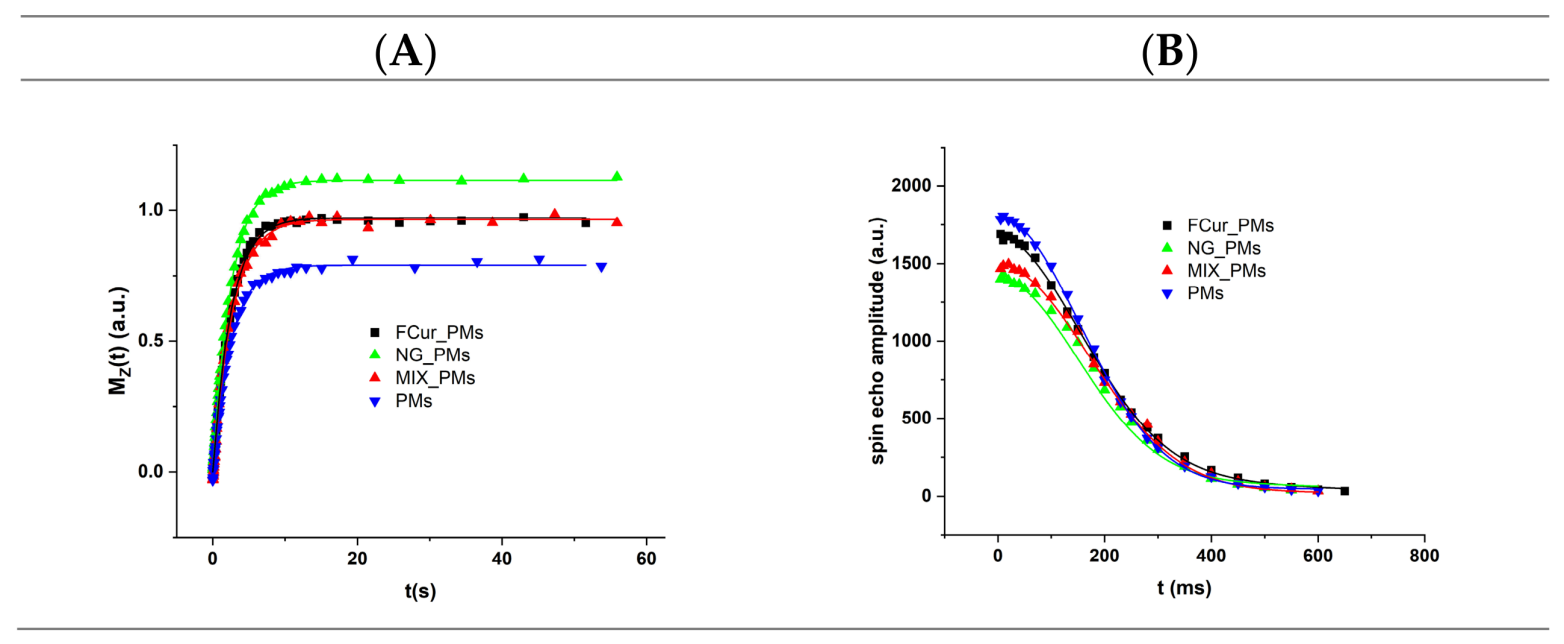
NMR relaxation analysis of micellar formulations: (**A**): Time-dependent recovery of longitudinal magnetisation (*T*_1_ relaxation) for all micellar formulations; (**B**): Time-dependent decay of transverse magnetisation (*T*_2_ relaxation) for all micellar formulations.

Representative magnetisation recovery curves and transverse relaxation decay profiles obtained for PM systems are presented in Figure 9.

The spin-lattice relaxation times determined for all formulations remained within a relatively narrow range (2.46–2.69 s), suggesting that the incorporation of active compounds does not substantially affect the overall molecular dynamics of the polymeric assemblies on the *T*_1_ timescale. The recovery curves were adequately described by a single-exponential model, indicating a single dominant relaxation environment on the *T*_1_ timescale.

In contrast, transverse relaxation analysis revealed heterogeneous dynamic behaviour in the micellar formulations. The spin-echo decay curves were fit using a two-component model comprising Gaussian and Lorentzian contributions, corresponding to more constrained and dynamically averaged molecular domains, respectively. The Gaussian phase dominated across all investigated formulations, indicating that most polymer segments exhibited restricted molecular motion, whereas the Lorentzian phase reflected more mobile regions within the micelles.

The determined *T*_1_ and *T*_2_ relaxation parameters for all investigated micellar formulations are summarised in Table 6. The pure PMs exhibited the highest *T*_2*L*_ value (1342 ms), indicating the greatest molecular mobility within dynamically averaged domains. In contrast, the incorporation of active compounds reduced the mobility of these regions to varying degrees, most likely due to drug–polymer interactions. The mixed MIX-PMs formulation exhibited relatively high *T*_2*L*_ values (847 ms) compared with systems containing single active compounds, suggesting enhanced motional averaging and increased dynamic homogeneity within the mixed formulation.

Overall, the obtained results indicate that incorporation of NG and FCur affects local molecular mobility and dynamic heterogeneity within Soluplus^®^-based micellar systems without causing large-scale structural rearrangements of the polymeric assemblies.

#### 3.5.3. Physicochemical Properties and Molecular Dynamics of Lyophilised Soluplus^®^ Micellar Systems

Due to their favourable physicochemical properties, PMs were selected for further studies on the effects of freeze-drying, to assess whether the process could alter their physicochemical properties and molecular dynamics.

Following lyophilisation and reconstitution, all investigated micellar systems retained nanoscale particle dimensions (below approximately 105 nm) and relatively low PDI values (below 0.2), suggesting that the freeze-drying process did not induce substantial aggregation or destabilisation of the polymeric assemblies (Table 7).

The zeta potential values remained slightly negative in all systems studied, as observed prior to freeze-drying, indicating that the micelles’ physicochemical characteristics were preserved. Importantly, lyophilisation had only a minor impact on drug retention, as the encapsulation efficiency of FCur and NG remained essentially unchanged in the single-drug micellar formulations. In the mixed system, only a negligible decrease in the amount of NG was observed (from 95.0% to 88.8%), suggesting that the freeze-drying process did not substantially compromise micellar integrity or cause compound degradation.

Since PMs were further subjected to lyophilisation, additional temperature-dependent ^1^H NMR relaxometry studies were performed to investigate how freeze-drying affects local molecular mobility and dynamic organisation within the resulting amorphous polymeric systems.

**Figure 10:**
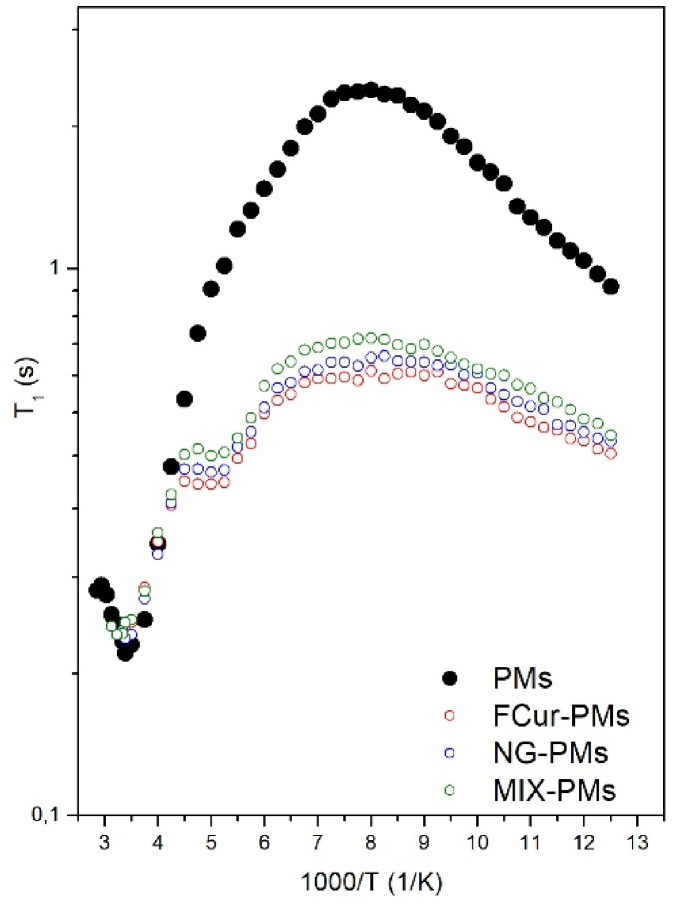
Temperature dependence of the spin–lattice relaxation times (*T*_1_) for lyophilised polymeric systems containing NG, FCur, and their mixture.

The temperature dependence of the spin-lattice relaxation times *T*_1_ plotted as a function of 1000/T, revealed similar relaxation behaviour for all investigated systems in the high-temperature region, suggesting dominance of common fast local motions associated with the Soluplus^®^ matrix. Differences between the investigated systems became apparent at lower temperatures, where a broad *T*_1_ maximum is observed (Figure 10). The highest *T*_1_ maximum detected for PMs indicates less efficient spin–lattice relaxation in the pure polymer matrix. In contrast, the lowered and broadened *T*_1_ maxima observed for NG-PMs, FCur-PMs, and MIX-PMs suggest the appearance of additional relaxation pathways resulting from interactions between the active compounds and the Soluplus^®^ matrix, as well as increased dynamic heterogeneity of the lyophilized systems [88,89].

The experimental temperature dependences were analysed using a multi-process Bloembergen–Purcell–Pound (BPP) model assuming thermally activated molecular motions. Fitting Equations (4) and (5) to the experimental data enabled determination of the activation parameters associated with the assumed molecular motions, namely the activation energy *E_a_* and the correlation time *τ_c_* (Figure S1; Table 8). An important observation was that pure PMs required only three motional processes to describe their relaxation behaviour, whereas all drug-containing formulations required four distinct motional contributions. This finding indicates the emergence of additional dynamically heterogeneous domains following incorporation of the active compounds. This observation suggests that the presence of NG, FCur, or their combination increases the complexity of local molecular dynamics within the amorphous polymer phase. Analysis of the activation parameters obtained from the ^1^H NMR relaxation data indicates that the molecular dynamics of the investigated systems are governed mainly by local molecular motions and secondary β-relaxation processes. The activation energies determined for the first and second motional processes are in the range of approximately 10–20 kJ mol^−1^, which is characteristic of restricted local motions of side groups and local conformational rearrangements in amorphous polymers. Similar activation energies associated with local side-chain dynamics and β-relaxation processes have been previously reported in NMR relaxometry studies of polymeric systems [90,91].

For pure PMs, three motional processes were identified. The first two processes, characterised by activation energies of 14.7 and 11.9 kJ mol^−1^, are most likely associated with restricted local motions of PVAc and PVCL side groups and local rearrangements of PEG segments. The third process, exhibiting a very low activation energy of 1.7 kJ mol^−1^, can be attributed to fast local motions such as methyl-group rotations originating primarly from the acetate moieties of PVAc segments. The low activation energy together with the short correlation times, indicates highly localised molecular dynamics.

The incorporation of NG into the Soluplus^®^ matrix leads to an increase in the activation energies of the first three motions, suggesting restricted local molecular mobility caused by drug–polymer interactions. NG contains hydroxyl groups capable of forming hydrogen bonds with carbonyl groups of PVCL and PVAc, as well as with the ether oxygen atoms of PEG chains. Such interactions may locally rigidify the amorphous structure and reduce the mobility of polymer side groups. Similar effects of molecular immobilisation in amorphous drug–polymer dispersions have been widely described in the literature [92].

An even stronger effect was observed for FCur-PMs formulation. The incorporation of FCur increased the activation energy of the third motional process from 1.7 kJ mol^−1^ in PMs to 9.7 kJ mol^−1^, indicating strong suppression of fast local motions. Due to its rigid aromatic structure and hydrophobic character, FCur may interact with Soluplus^®^ through hydrogen bonding, dipolar interactions, and hydrophobic interactions, resulting in enhanced local immobilization of the polymer matrix and restriction of fast local molecular motions.

The MIX-PMs system exhibits dynamic behaviour intermediate between that of the NG-PMs and FCur-PMs. The relatively high activation energy of the second process (18.7 kJ mol^−1^) suggests the formation of locally rigid domains resulting from interacions between the active compounds and the Soluplus^®^ matrix. At the same time, the lower activation energy of the third process compared with FCur-PMs may indicate partial competition between NG and FCur for interaction sites within the polymer matrix.

An important feature of all drug-containing systems is the appearance of an additional fourth motional process, absent in pure Soluplus^®^. This process is characterised by very low activation energies (~1 kJ mol^−1^) and high Δ*M*_2_ values, suggesting the presence of additional fast local proton fluctuations within heterogeneous amorphous drug–polymer domains formed during lyophilisation. The large Δ*M*_2_ contribution indicates that this process contributes to the overall proton relaxation mechanism. The emergence of this additional relaxation pathway further supports the increased dynamic heterogeneity of the multicomponent amorphous systems [85].

Taking together, the determined activation parameters demonstrate that the incorporation of NG, FCur, and their mixture modifies the local molecular dynamics of the Soluplus^®^ matrix through specific drug–polymer interactions and formation of heterogeneous amorphous domains. These results confirm that ^1^H NMR relaxation studies is a highly sensitive tool for probing local molecular mobility and dynamic heterogeneity in lyophilised polymer-based drug delivery systems.

## 4. Conclusions

In the present study, two structurally distinct nanocarriers, Soluplus^®^-based polymeric micelles and POPC:DOTAP liposomes, were successfully developed for the encapsulation of FCur, NG, and their combination. They were comparatively characterised with respect to their physicochemical properties, encapsulation efficiency, molecular dynamics, and in vitro anticancer activity. The obtained results demonstrated pronounced carrier-dependent differences in particle size distribution, dispersity, surface charge, and local molecular organisation. PMs formed relatively small and homogeneous nanoparticles with low polydispersity and weakly negative zeta potential, whereas LPs exhibited larger particle sizes together with strongly positive surface charge, reflecting distinct stabilisation mechanisms and structural organisation within the investigated nanocarriers.

Both carrier systems enabled efficient encapsulation of the investigated compounds, with particularly high loading efficiency observed for NG-containing formulations. Furthermore, lyophilised micellar systems retained favourable physicochemical properties after rehydratation.

A biological in vitro study demonstrated that nanoencapsulation substantially influenced the cytotoxicity of the tested compounds in a carrier-dependent manner. Among the evaluated formulations, liposomal systems, particularly FCur-LPs and MIX-LPs, demonstrated the highest in vitro cytotoxic activity. Nevertheless, this increased cytotoxicity was not associated with a clear preference for cancer cells, but rather reflected a general enhancement of cytotoxic effects under the experimental conditions. In contrast, polymeric micelles with FCur and MIX-PMs showed a more favourable cytotoxicity profile, maintaining low toxicity toward normal cells while retaining activity particularly against one of the tested cancer cell lines (5637).

^1^H NMR relaxometry revealed pronounced differences in molecular organisation and mobility between the investigated carrier systems. Liposomes were characterised by more restricted molecular motions associated with phospholipid bilayer packing, whereas micelles exhibited greater mobility within the carrier matrix. Temperature-dependent studies of lyophilised micelles demonstrated that the incorporation of active compounds altered dynamic behaviour of the amorphous polymer phase. The emergence of an additional relaxation process in all drug-containing formulations suggested the presence of dynamically distinct molecular environments within the Soluplus^®^ matrix.

In summary, the obtained results demonstrate that the nanocarrier type strongly influences the physicochemical and biological behaviour of the encapsulated compounds. The findings suggest that the obtained liposomal systems provide enhanced in vitro cytotoxic activity, whereas micellar formulations may offer a more favourable biological profile due to their lower cytotoxicity toward non-malignant cells.

From the perspective of the rational design of nanocarriers, these results indicate that the carrier architecture, surface charge, and local molecular organisation should be considered collectively, rather than as independent parameters of the formulation. Adapting these properties may improve the balance between effective drug delivery, anticancer activity, and selectivity towards tumor cells. These findings provide a framework for the development of future nanocarrier systems designed for hydrophobic anticancer compounds.

## Figures and Tables

**Table 2:** Encapsulation efficiency of FCur and NG compounds in micelles, and liposomes.

Sample	c [µM]	EE [%]
**Micelles**		
FCur-PMs	72.1	72.32 ± 4.84
NG-PMS	96.9	97.09 ± 1.13
MIX-PMs		
FCur	36.1	72.78 ± 5.55
NG	47.4	95.00 ± 2.51
**Liposomes**		
FCur-LPs	74.5	74.63 ± 3.23
NG-LPs	98.8	98.95 ± 1.34
MIX-LPs		
FCur	40.0	80.13 ± 1.42
NG	48.7	97.33 ± 4.72

**Table 4:** Comparison of liposomes and polymeric micelles as drug delivery systems.

Features	Liposomes	Polymeric Micelles
Drug loading	Encapsulate both hydrophilic (aqueous core) and hydrophobic (lipid bilayer) drugs [67,68,69].	Primarily designed for poorly water-soluble (hydrophobic) drugs encapsulated within the polymeric core [51,70,71].
Mechanism of drug delivery	Membrane interaction and endocytosis; cationic lipids (e.g., DOTAP) enhance electrostatic interaction with cell membranes [72,73,74].	Improve drug solubility and stability in biological environments prior to reaching the target site; cellular uptake occurs via micelle-mediated transport and/or endocytosis, depending on the formulation [70,75].
Intrinsic biological activity of the carrier	Possible, particularly for cationic lipids (e.g., DOTAP, DOTMA) [72,74,76].	Generally exhibit minimal intrinsic biological activity [70,71].
Typical engineering strategy	Lipid composition, PEGylation, targeting ligands (folate, antibodies, RGD peptides, hyaluronic acid) and stimuli-responsive lipids to enhance cellular uptake, targeting and controlled drug release [69,73,77].	Amphiphilic polymer selection (PEG-PLA, PEG-PCL, PEG-PLGA, PEG-PGlu) or mixed micelles (Soluplus/TPGS, Soluplus/Solutol HS15) to improve drug loading, solubility, stability, and pharmacokinetics [70,78,79].
Representative formulations and clinical translation	Several liposomal formulations have already received clinical approval and are in clinical use, including Doxil^®^, AmBisome^®^, Onivyde^®^, and Vyxeos^®^. Other advanced liposomal systems, such as ThermoDox^®^ and EndoTAG-1, remain under clinical investigation [80,81].	Approved formulation includes Genexol PM^®^ whereas investigational systems include NK105 and NC-6004 [70,82].

**Table 5:** The obtained values of the spin-lattice *T*_1_ and the spin-spin *T*_2_ relaxation times in the laboratory frame.

Sample	*T*_1_ (s)	$M_{0G}$ (%)	*T*_2*G*_ (ms)	$M_{0L}$ (%)	*T*_2*L*_ (ms)
FCur-LPs	2.73	65	228	35	252
NG-LPs	2.71	98	238	2	337
MIX-LPs	2.76	88	243	12	482
LPs	2.64	90	228	10	430

**Table 6:** Proton spin-lattice *T*_1_ and spin-spin *T*_2_ relaxation times in the laboratory frame.

Sample	*T*_1_ (s)	$M_{0G}$ (%)	*T*_2*G*_ (ms)	$M_{0L}$ (%)	*T*_2*L*_ (ms)
FCur-PMs	2.50	81	222	19	342
NG-PMs	2.46	89	211	11	644
MIX-PMs	2.69	97	238	3	847
PMs	2.52	96	216	4	1342

**Table 7:** Physicochemical properties and encapsulation efficiency of lyophilised micellar formulations.

Sample	MDD ± SD [nm]	PDI ± SD	ZP ± SD [mV]	c [µM]	EE [%]
Lyophilizates					
FCur-PMs	103.70 ± 3.68	0.167 ± 0.07	−5.96 ± 2.64	74.3	74.34 ± 0.88
NG-PMs	77.75 ± 12.07	0.178 ± 0.02	−4.16 ± 2.81	101.6	101.77 ± 2.11
PMs	99.43 ± 15.80	0.151 ± 0.06	−7.49 ± 3.34		
MIX-PMs					
FCur	105.40 ± 1.62	0.169 ± 0.01	−1.96 ± 1.29	37.4	74.80 ± 4.24
NG				42.2	88.82 ± 1.33

**Table 8:** Activation parameters of molecular motions for the investigated lyophilised micellar systems.

Samples	I Motion			II Motion			III Motion			IV Motion		
	*τ*_0_ (s)	*Ea* kJ mol^−1^	Δ*M*_2_ Gs^2^	*τ*_0_ (s)	*Ea* kJ mol^−1^	Δ*M*_2_ Gs^2^	*τ*_0_ (s)	*Ea* kJ mol^−1^	Δ*M*_2_ Gs^2^	*τ*_0_ (s)	*Ea* kJ mol^−1^	Δ*M*_2_ Gs^2^
PMs	9.9 × 10^−12^	14.7	1.1	1.01 × 10^−12^	11.9	0.1	2.18 × 10^−11^	1.7	1.5	—	—	—
FCur-PMs	6.0 × 10^−12^	16.3	0.9	6.43 × 10^−13^	14.1	0.3	9.95 × 10^−13^	9.7	0.1	1.32 × 10^−11^	0.9	17.6
NG-PMS	3.2 × 10^−12^	17.4	0.9	1.68 × 10^−13^	16.2	0.2	9.27 × 10^−12^	7.6	0.1	1.11 × 10^−11^	1.0	16.6
MIX-PMs	1.5 × 10^−11^	14.3	0.9	2.83 × 10^−14^	18.7	0.2	2.09 × 10^−11^	6.8	0.1	1.18 × 10^−11^	1.0	15.8

## Data Availability

The data presented in this study are available within the article and its Supplementary Materials.

## Supplementary Materials

The following supporting information can be downloaded at: https://www.mdpi.com/article/10.3390/nano16150920/s1, Table S1. Calibration curve parameters for the determination of FCur and NG by HPLC with gradient elution. Table S2. Representative physicochemical characteristics of liposomal and polymeric micellar formulations obtained during formulation optimization. Figure S1. The temperature dependence of the spin-relaxation time T1 for NG-PMs. FCur-PMs.
