# Supplementary material for: Liposomal and Micellar-Based Nanoformulations of Fluorinated Curcumin Derivative and Naringenin—Comparative Studies
Source: Nanomaterials (Basel). 2026 Jul 27;16(15):920. doi: 10.3390/nano16150920 (PMC13468390; doi:10.3390/nano16150920)
Supplement: Supplementary file 1 [file nanomaterials-16-00920-s001.zip › nanomaterials-4427183-supplementary.pdf]

nanomaterials

MDPI

Supplementary Materials

Liposomal and Micellar-Based Nanoformulations of Fluorinated Curcumin Derivative and Naringenin—Comparative Studies

Joanna Kuzminska[1,2,*], Agnieszka Sobczak[1,*], Ludwika Piwowarczyk[1], Violetta Krajka–Kuźniak[3], Rafał Pietrzyk[4], Mikołaj Baranowski[4], Paweł Bilski[5], Aneta Woźniak-Braszak[4], Tomasz Goslinski[6], Anna Jelińska[1]

[1] Poznan University of Medical Sciences, Chair and Department of Pharmaceutical Chemistry, Rokietnicka 3, 60-806 Poznan, Poland, lpiwowarczyk@ump.edu.pl (L.P.); ajelinska@ump.edu.pl (A.J.)
[2] Poznan University of Medical Sciences, Doctoral School, Bukowska 70, 60-812 Poznan, Poland;
[3] Poznan University of Medical Sciences, Chair and Department of Pharmaceutical Biochemistry, Rokietnicka 3, 60-806 Poznan, Poland, vkrajka@ump.edu.pl
[4] Functional Materials Physics Division, Faculty of Physics and Astronomy, Adam Mickiewicz University, Uniwersytetu Poznanskiego 2, 61-614 Poznan, Poland, rafal.pietrzyk@amu.edu.pl (R.P.); mikbar@amu.edu.pl (M.B.); aneta.wozniak-braszak@amu.edu.pl (A.W.-B.)
[5] Department of Experimental Physics of Condensed Phase, Faculty of Physics and Astronomy, Adam Mickiewicz University, Uniwersytetu Poznanskiego 2, 61-614 Poznan, Poland, bilski@amu.edu.pl
[6] Poznan University of Medical Sciences, Chair and Department of Chemical Technology of Drugs, Rokietnicka 3, 60-806 Poznan, Poland, tomasz.goslinski@ump.edu.pl
* Correspondence: jkuzminska@ump.edu.pl; asobczak@ump.edu.pl

Table S1. Calibration curve parameters for the determination of FCur and NG by HPLC with gradient elution

Compound	Statistical evaluation	
	$y = ax + b$	$y = ax$
FCur	$a = 98.87 \pm 0.67$	$a = 98.72 \pm 0.60$
	$b = -1.34 \pm 4.15$	
	$S_a = 0.240$	$S_a = 0.214$
	$S_b = 1.496$	
	$S_y = 2.633$	$S_y = 2.355$
	$\lvert t_b \rvert = 0.897 < t_{0.05}(4)$	
	$t_{0.05}(6) = 2.447$	r = 0.999
	r = 0.999	
NG	$a = 34.84 \pm 0.33$	$a = 34.65 \pm 0.30$
	$b = -0.89 \pm 1.17$	
	$S_a = 0.113$	$S_a = 0.124$
	$S_b = 0.476$	
	$S_y = 0.890$	$S_y = 0.824$
	$\lvert t_b \rvert = 1.873 < t_{0.05}(6)$	
	$t_{0.05}(4) = 2.306$	r = 0.999
	r = 0.999	

a - directional coefficient of the straight line, *b* - coefficient of the directional form of the linear function, S_a - parameter standard error of a, S_y- parameter standard error of a, S_b- parameter standard error of b, r - linear correlation coefficient

Academic Editor: James C. L. Chow

Received: 24 June 2026
Revised: 14 July 2026
Accepted: 22 July 2026
Published: 27 July 2026

Copyright: © 2026 by the authors. Licensee MDPI, Basel, Switzerland. This article is an open access article distributed under the terms and conditions of the Creative Commons Attribution (CC BY) license.

Table S2. Representative physicochemical characteristics of liposomal and polymeric micellar formulations obtained during formulation optimization

Formulation	MDD [nm]	PDI	ZP [mV]	EE of FCur [%]
POPC:DOTAP 7:0.7	316.6	0.467	+27.40	36.11
POPC:DOTAP 8:0.5	215.5	0.451	+29.42	35.15
POPC:DOTAP 8:0.7	153.9	0.425	+31.32	36.44
POPC:DOTAP 8:0.8 extrusion	118.4	0.111	+41.47	7.14
Soluplus® 105.0 mg/mL	103.1	0.246	-1.83	36.11
Soluplus® 62.0 mg/mL	98.2	0.201	-1.92	69.84
Soluplus® 31.5 mg/mL 15 min mixing	81.28	0.196	-2.81	70.23
Soluplus® 31.5 mg/mL 20 min mixing	85.03	0.180	-2.13	71.34

https://doi.org/10.3390/nano16150920

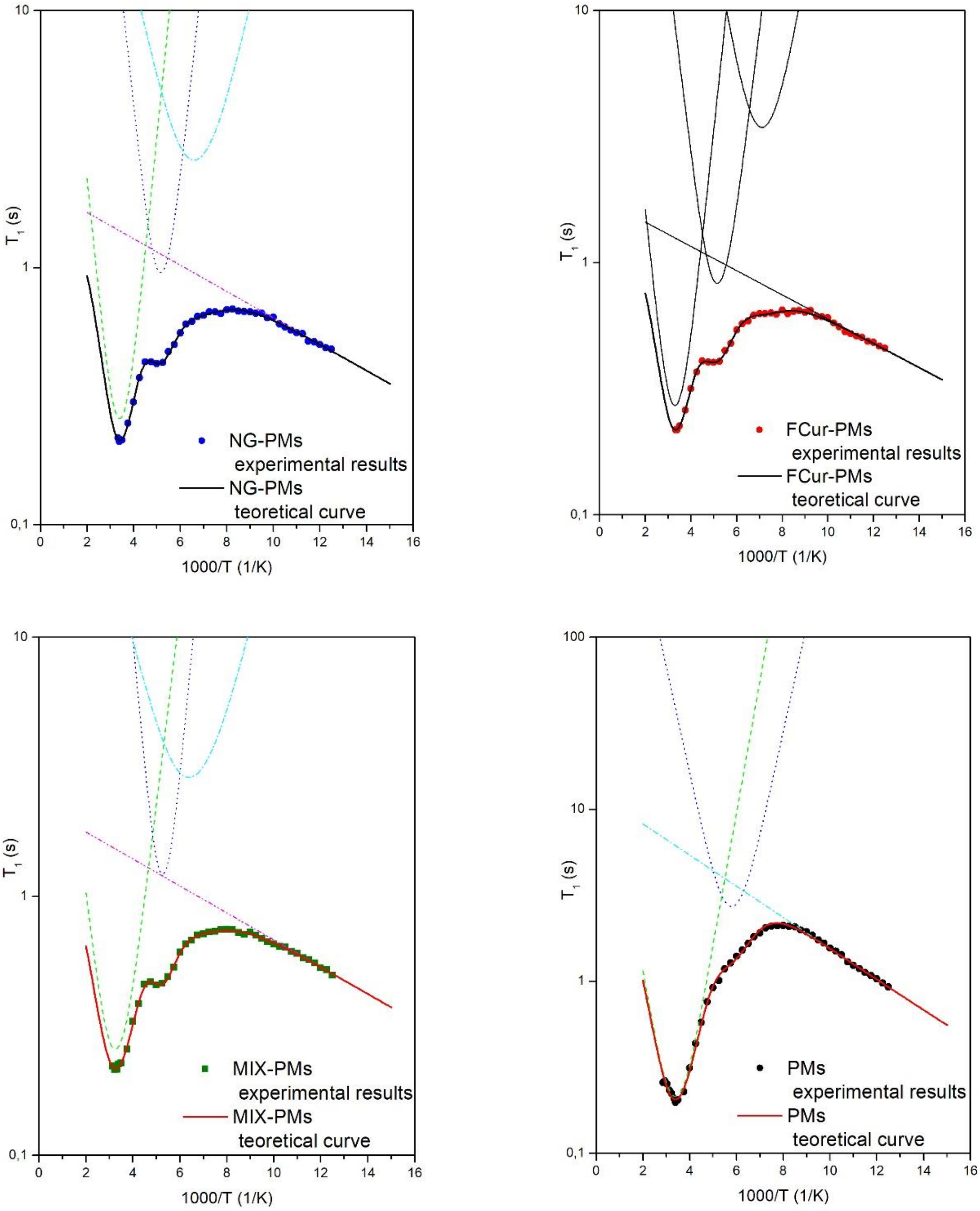

Figure S1. The temperature dependence of the spin-relaxation time T_1 for NG-PMs. FCur-PMs. MIX-PMs and PMs. The solid lines represents the best fits of Eq. 4 and Eq. 5 to the experimental data.

$$\frac{1}{T_1} = \frac{2}{3}\gamma^2 \sum \Delta M_{2k} \left(\frac{\tau_{ck}}{1+\omega_0^2\tau_{ck}^2} + \frac{4\tau_{ck}}{1+4\omega_0^2\tau_{ck}^2} \right) \quad \text{(S1)}$$

$$\tau_{ck} = \tau_0 exp \left(\frac{E_{ak}}{RT} \right) \quad \text{(S2)}$$

https://doi.org/10.3390/nano16150920
